# Energy contribution of *NOVA* food groups and the nutritional profile of the Brazilian rural workers' diets

**DOI:** 10.1371/journal.pone.0240756

**Published:** 2020-10-28

**Authors:** Monica Cattafesta, Glenda Blaser Petarli, Eliana Zandonade, Olívia Maria de Paula Alves Bezerra, Sandra Marlene Ribeiro de Abreu, Luciane Bresciani Salaroli

**Affiliations:** 1 Graduate Program in Collective Health, Federal University of Espírito Santo, Vitória, ES, Brazil; 2 Department of Family Medicine, Mental and Collective Health, Federal University of Ouro Preto, Ouro Preto, MG, Brazil; 3 Faculty of Sports, Research Center on Physical Activity, Health and Leisure, University of Porto, Porto, Portugal; Addis Ababa University, ETHIOPIA

## Abstract

We estimated the caloric contribution of minimally processed foods, processed culinary ingredients, processed foods and ultra-processed foods in Brazilian farmers' diets and their association with the nutritional profile of the diet. It is an epidemiological study of cross-sectional, analytical and quantitative design with 740 farmers adults of Southeastern Brazil. Food intake data were obtained by applying three 24-hour recalls and were classified according to the degree and purpose of processing. The largest caloric contribution came from the group of minimally processed foods (64.7%), followed by ultra-processed foods (17.7%), processed culinary ingredients (12.4%), and processed foods (5.2%). Individuals in the fourth quartile of caloric contribution from minimally processed foods showed lower energy consumption (β -0.16, *P*<0.001) and greater consumption of all 15 micronutrients analyzed. In contrast to what was identified for this food group, the higher caloric contribution from ultra-processed foods was associated with a greater caloric content of the diet (β 0.17, *P*<0.001) and lower consumption of all 23 analyzed nutrients. Therefore, the caloric contribution from the consumption of ultra-processed foods in the rural area is still lower than the national average. However, measures aimed at delaying isocaloric exchanges for the group of ultra-processed foods must be carried out, maintaining the local food culture, since this group had worse nutritional levels. In addition, incentives to the greater consumption of minimally processed foods should be carried out, due to their nutritional quality.

## Introduction

Agriculture is an integral part of our culture, as almost all of human history involves agrarian societies [1]. However, the way in which human beings produce and obtain food has undergone major changes over the years [2], changing the perception of food produced by farmers, which has come to be understood as a commodity, a form of subsistence and necessary work tool [1, 3, 4] for supply the agro-industrial market [1, 5].

The change in the food consumption of the populations also accompanied these modification in the form of food production and distribution, multiplying the availability of food [6], with foods increasingly displaced from their geographic roots, standardizing and homogenizing in various parts of the globe, offering products increasingly closer to the stage of consumption [7].

In this context, as of 2010, with the publication of studies by Monteiro et al. [8], research in the field of food, nutrition and health was based on the new classification of foods (*NOVA*—a name, not an acronym), that takes into account the extent and purpose of processing, the basis of the Food Guide for the Brazilian Population [9]. This methodology classifies foods and food products into four clearly distinct groups: *in natura* and minimally processed foods; processed culinary ingredients; processed foods; and ultra-processed foods [10].

This way of investigating eating habits is in line with the transition of food systems, since the consumption of ultra-processed foods has become dominant worldwide [10], with a high caloric contribution being demonstrated from these types of foods in developed countries such as United States of America (60.0%) [11], United Kingdom (54.3%) [12] and Canada (45.0%) [13]. But not unlike this profile, Brazil also showed a significant increase in the share of ready-to-eat products in its diet (from 23.0% to 27.8% of calories), due to the increase in the acquisition of ultra-processed products (from 20.8% to 25.4% between 2002–2003 and 2008–2009) and a significant reduction in the participation of minimally processed foods and culinary ingredients [14]. Moreover, 20.4% of the energy contribution of the Brazilian diet comes from ultra-processed foods [15].

Some studies have identified a lower nutritional quality of ultra-processed foods [15–29], however, it seems that the acquisition of this type of food is still less in rural areas [30] and there are no studies available, so far, on the evaluation of the nutritional profile of the farmers' diet related to the degree and purpose of food processing, even though it has already been identified that this is a group with high cardiovascular risk [31], multimorbidity [32] and also adherence to an industrialized dietary pattern [33]. Because of the “agrarian myth”, it is believed that the traditional agricultural population enjoys a fully healthy life, with excellent eating habits [34]. However, epidemiological studies demonstrate changes in rural eating habits, share many similarities with their urban peers [33, 35–39]. Although farmers have options to obtain food through their own plantations or community sharing, there are also food deserts in remote agricultural areas [40, 41], with high costs for healthy foods [42], difficult access due the long distances or lack of means of transport, and difficulty in adequate storage of fresh and healthy products [41, 43]. Furthermore, the low education of these workers can also influence their income and healthy food choices [41] or the lack of understanding of the food produced as food for self-consumption, recognizing them only as merchandise and source of income [3]. In addition, rural labor structures, in which the whole family is involved or the long distances required to farm, in some cases, can lead to a lack of time for preparation of healthy meals [41]. Thus, the objective of this study was to estimate the caloric contribution of minimally processed foods, processed culinary ingredients, processed foods and ultra-processed foods in Brazilian farmers' diets and their association with the nutritional profile of the diet.

## Materials and methods

### Study design

This is an epidemiological study of cross-sectional, analytical and quantitative design developed in the municipality of Santa Maria de Jetibá, located in the highlands of the state of Espírito Santo, Southeastern Brazil. This study integrates, in larger scope, the study “Health condition and associated factors: a study in farmers of Espírito Santo—AgroSaúdES”.

The representative sample of male and female farmers exposed here met the following inclusion criteria: adults 18–59 years old, non-pregnant, who had agriculture as their main source of income and were in full employment for at least six months. Individuals who did not meet these criteria were excluded. To identify eligible farmers, we used data available in the records of individuals and families conducted by the Family Health Strategy teams, responsible for covering 100% of the eleven health regions of the municipality.

### Sampling

7,287 farmers were identified from a total of 4,018 families. About this population universe, we calculated a minimum sample of 708 farmers, considering a sampling error of 3.5%, 95% confidence interval and prevalence of 50% to maximize sample. In order to reach the minimum sample and considering possible losses, recruitment included 806 individuals.

To define the sample universe one list was built with the survey of the registration of individuals and families by the Community Health Agents, through the data available in the family register used by the Family Health Strategy teams. This register covers 100% of the eleven health regions of the municipality. The participants were selected by stratified draw, proportionality the number of families per health region, in order to respect proportionality among the eleven regions. In families with more than one eligible, only one individual was drawn, avoiding thus the interdependence of information. In cases of refusal of participation or non-attendance in data collection, a new participant in the waiting list of the lottery was called, respecting the sex and region of origin of the dropout.

### Data collection

Data collection took place between December 2016 and April 2017 in the facilities of the municipal health units of the Family Health Strategy teams. A semi-structured questionnaire containing questions about socioeconomic, occupational, lifestyle and food consumption characteristics was applied [44].

### Food consumption data collection

Food consumption data were obtained by applying three 24-hour recalls (R24h) during the interview, two days of the week and one day of the weekend within 15 days after the first R24h in the return interviews. In order to obtain greater accuracy of the portions consumed, photo albums were used to facilitate the identification and quantification of the consumed items. Data were collected from 790 farmers, but 27 individuals were excluded since they underwent only one R24h and 23 since they underwent only two (6.3% loss), resulting in a final sample of 740 farmers. As such, the total lied above the minimum sample of 708 farmers, therefore, being then representative of the total population.

The nutritional composition of the R24h was performed using the software AvaNutri 4.1 (Avanutri Equipamentos de Avaliação Ltda, Três Rios, RJ, Brazil) in which the Brazilian Table of Food Composition [45] was selected for extraction of nutritional information. Information from the manufacturer or from standard recipes was used for the registration of typical regional foods that were not available at a table and possible dietary supplements were registered. After the registration of the food and acquiring the calories, no exclusion was performed due to extremes in energy consumption [46].

After obtaining the values of each R24h, the analysis of the attenuation was performed using the PC-SIDE software (Department of Statistics, Iowa State University, Iowa, EUA), which follows the methodology of Nusser et al. [47]. Then, the adjustment for energy by the residual method was carried out, which corrects the estimates of nutrients by total energy intake [46].

### Classification of food according to the degree and purpose of processing

The foods consumed by farmers in the three R24h were listed (n = 355 food items). These foods were classified according to *NOVA*, that is, in four groups: *in natura* and minimally processed foods; processed culinary ingredients; processed foods; and ultra-processed foods [8, 48]. This classification takes into account the extent and purpose of foods processing, being food processing classified as any physical, biological and chemical process that occurred after harvesting or separating food from nature and before it was subjected to culinary preparation in the kitchens of houses or restaurants, or before its consumption when are processed products fully ready for consumption [48]. The methods used by farmers and producers in growing plants and raising animals are not considered in such a classification [8].

*In natura* and minimally processed foods are edible parts of plants (seeds, fruits, leaves, stems, roots) or animals (muscles, viscera, eggs, milk) and also mushrooms and algae after their separation from nature that have undergone minimal processes, such as cleaning and conditioning. Processed culinary ingredients are substances extracted directly from group 1 foods or from nature and consumed as items of culinary preparations. Processed foods are products manufactured by adding salt or sugar and, eventually, oil, vinegar or another group 2 substance to a group 1 food (e. g. canned or bottled whole vegetables and legumes preserved in brine, whole fruits preserved in syrup, tinned fish preserved in oil, some types of processed meat and cheese). Most of these products contain two or three ingredients. Finally, ultra-processed foods are industrial formulations typically made with five or more ingredients. These ingredients often include substances and additives used in the manufacture of processed foods, unusual substances in culinary preparations, additives whose function is to simulate sensory attributes of group 1 foods or culinary preparations of these foods, or to hide undesirable sensory attributes in final product (e. g. breakfast cereals, cake mixes, instant soups and noodles, many types of packaged breads, fatty or salty snack products, sugared drinks, candies (confectionery), margarines, cts). Group 1 foods represent a reduced proportion or are not even present in the list of ingredients of ultra-processed products [8, 9, 48].

Each food item was classified according to how it was consumed, as reported by farmers. In the R24h interview, the items combined/prepared together "by hand", whenever possible, were disaggregated for classification. The products/meals purchased ready were classified directly to the item consumed and the recipes made "by hand" without the possibility of disaggregating the ingredients were categorised according to the main constituent ingredient (for example: homemade breads, subgroup of flours; vinaigrette salad and soups, vegetables subgroup) [49].

After the classification of food items in each *NOVA* group, the calories from each food group and subgroup were added. Then, the caloric contribution of each food group to daily energy consumption was calculated as follows: Caloric contribution of the food group assessed = Calories from food consumption in this assessed group x 100 ÷ total gross calorie. The use of this indicator of caloric contribution of each food group (given in % of contribution) is more recommended than the gross energy of each food group, since it minimizes the differences in the total energy consumed (due to the differences in energy requirements of individuals), assesses the quality of the diet and not the quantity consumed and also makes it possible to assess the extent to which there is an exchange of culinary preparations for ultra-processed foods [8, 19, 23].

### Nutritional profile of the diet according to nutrient consumption

The nutritional profile of the diet was evaluated using the following markers: energy consumption (kcal and kJ), energy density (kcal/g), percentage of calories from macronutrients (carbohydrates, proteins, lipids, saturated fatty acids–SFA–and polyunsaturated fatty acids–PUFA), cholesterol, fiber, vitamin A, vitamin B1, vitamin B2, vitamin B3, vitamin B6, vitamin B9, vitamin C, vitamin E, calcium, iron, phosphorus, potassium, sodium, selenium, zinc and copper (expressed in g, mg or μg per 1000 kcal).

In addition, the consumption of nutrients by each subject was evaluated according age- and sex-specific current recommendations in “within the recommended”, when it was within the recommended range, and “inadequate intake”, when it was above the tolerable upper intake or below the intake's recommendation (S1 Table). The energy density was calculated for the solid fraction of the diet, which corresponds to the sum of calories from solid foods divided by the quantity in grams of these foods and classified according to the World Cancer Research Fund & American Institute for Cancer Research [50]. The percentage of total energy intake of carbohydrate, protein, lipid and PUFA followed the World Health Organization (WHO) recommendations published in 2003 [51], as well as the consumption of cholesterol and fibers. The percentage of SFA followed the WHO indications published in 2018 [52], as well as the consumption of potassium and sodium. The consumption of vitamins and minerals followed the recommendations of the Dietary Reference Intakes (DRI) of the Food and Nutrition Board (FNB) [53], using the values of Estimated Average Requirement (EAR) until the Tolerable Upper Intake Level (UL).

### Statistical analysis

The normality of the variables was assessed by the Shapiro-Wilk test. To describe the study variables, the median and mean was used as a measure of central tendency, and the interquartile range and standard deviation as a dispersion measure for continuous variables. The variables of food consumption (*NOVA*) were investigated in their continuous values and also categorized in quartiles.

To analyze the association between a quantitative and a qualitative variable, due to the abnormality of the variables, the Mann-Whitney U test was used. When the qualitative variable had three or more categories, the Kruskal-Wallis test and the Mann-Whitney U test were performed two by two to identify the differences. Missing data were maintained due to low data loss, different number of individuals in each variable were reported in the table captions.

The linear regression model was used to analyze the nutritional profile of the diet according to the caloric contribution of the *NOVA* groups (first quartile *versus* fourth quartile). The variables that had statistical significance with the food groups of up to 20% (i.e. *P*<0.02) in the binary analyzes (presented in S2 Table) were used as adjustment variables in the multiple models, that could include sociodemographic, labor, and lifestyle variables.

Among the sociodemographic variables were evaluated sex, age group (“up to 29 years”, “30 to 39 years”, “40 to 49 years” and “50 years or more”), marital status (“single”, “married/living with a partner” and “divorced/separated/widowed”), race/color (“white” and “non-white”), schooling (“less than 4 years”, “4 to 8 years” and “more than 8 years”), land bond (“owner” and “non-owner”) and socioeconomic class (“A or B”, “C” and “D or E”), according to the Criteria of Economic Classification Brazil, used in national studies to estimate socioeconomic classes according to the purchasing power of individuals and families, projecting the average monthly gross family income [54]. Labor variables were investigated by questioning the current type of production (“conventional” and “non-conventional”), the type of worked crops categorized into “temporary only”, “permanent only” and “temporary and permanent”, according to criteria of the Brazilian Institute of Geography and Statistics [55] and the workload (hours/week) (“less than or equal to 40 hours” and “more than 40 hours”). Lifestyle variables included alcohol consumption, categorized as “non-drinking” and “drinking”; smoking, assessed according to the Smoker Approach and Treatment Consensus and categorized as “non-smoker” and “current and past smoker”; practice of physical activity extra-field (“yes” or “no”); and screen time obtained by the sum of daily activities for television, video game and computer/cell phone, divided by the days of the week, classified as “no sedentary leisure” when <2 hours/day and “with sedentary leisure” when ≥ 2 hours/day [56].

We used the Enter variable selection method. We also tested the assumptions of absence of multicollinearity (tolerance >0.1 and variance inflation factor <10), minimum sample size for the number of model variables (>20 individuals per model variable and >5 cases in each category of variables), absence of outliers (absence of standardized residues >±3 standard deviations; up to 1% of standardized residues between ±2.5 and 3 standard deviations; and up to 5% of standardized residues between ±2.0 and 2.5 standard deviations, Cook's distance <1, and DFBeta <1), residues normally distributed (Durbin-Watson 1.5 to 2.5) and presence of homoscedasticity.

For all analyses, the level of significance adopted was α<5% and these were performed using the statistical software IBM SPSS Statistics for Windows version 22.0 (IBM Corp, Armonk, NY, EUA).

### Ethical standards disclosure

This study was conducted according to the guidelines laid down in the Declaration of Helsinki and all procedures involving research study participants were approved by the Research Ethics Committee of the Health Sciences Center of the Federal University of Espírito Santo (Ufes) under number 1,856,331 (CAAE 52839116.3.0000.5060). Written informed consent was obtained from all subjects.

## Results

Of the 740 farmers evaluated, 51.5% (n = 381) was men and 48.5% (n = 359) was women. Most of the evaluated farmers were married or living with a partner, were in poor socioeconomic class and had low schooling (67.7% with less than 4 years of schooling) (Table 1).

**Table 1:** General characteristics of study population.

Variables	n	%
**Sex**		
Male	381	51.5
Female	359	48.5
**Age group**		
Up to 29 years	201	27.2
30 to 39 years	218	29.5
40 to 49 years	183	24.7
50 years or more	138	18.6
**Marital status**		
Single	56	7.6
Married/living with a partner	638	86.2
Divorced/separated/widowed	46	6.2
**Race/color**		
White	669	90.4
Non-white	71	9.6
**Socioeconomic class**		
A or B	56	7.6
C	376	50.8
D or E	308	41.6
**Schooling**		
Less than 4 years	501	67.7
4 to 8 years	161	21.8
More than 8 years	78	10.5

n = 740.

The average caloric consumption of farmers was 2,561.0±883.1 kcal (Table 2), whom the group of minimally processed foods was the one that presented the greatest caloric contribution of this diet (64.7%), followed by ultra-processed foods (17.7%), processed culinary ingredients (12.4%), and processed foods (5.2%). The food items that contributed most energetically to the composition of the minimally processed food group were flours, meats, rice, and beans. The largest contributors to the processed foods group were breads, cookies, cakes and pastries. In the ultra-processed food group, consumption of pasta, bread, biscuits, cereals and industrialized sweets and sugary drinks stood out.

**Table 2:** Total calories and caloric contribution of *NOVA* groups and subgroups to the food consumption of Brazilian farmers.

*NOVA*’s groups and subgroups	Total calories from consumption by *NOVA* groups and subgroups		Caloric contribution of *NOVA* groups and subgroups in daily energy consumption						
			Total		Sex				
					Male		Female		p-value*
					(n = 381)		(n = 359)		
	Mean	SD	Mean	SD	Mean	SD	Mean	SD	
	(kcal)		(%)		(%)		(%)		
**Minimally processed**	**1670.9**	**716.2**	**64.7**	**13.1**	**65.3**	**12.8**	**64.2**	**13.4**	0.327
Flours (corn, wheat and cassava)	597.4	519.0	22.1	14.4	21.6	14.8	22.6	14.0	0.229
Red meat	257.4	295.2	9.7	9.5	11.2	10.2	8.1	8.4	**<0.001**
Poultry meat	212.6	203.9	8.5	7.0	8.5	6.9	8.6	7.1	0.849
Rice	169.2	97.4	6.9	3.9	7.1	3.8	6.7	3.9	**0.047**
Beans	150.7	108.5	6.0	3.9	6.5	4.2	5.4	3.6	**<0.001**
Potatoes, roots and tubers	70.6	80.9	2.9	3.0	2.9	3.3	2.8	2.6	0.441
Fruit^a^	53.5	77.7	2.2	3.1	1.7	2.7	2.7	3.5	**<0.001**
Eggs	41.5	65.3	1.7	2.7	1.7	2.8	1.6	2.7	0.742
Vegetables and green condiment^b^	33.2	40.4	1.4	1.7	1.1	1.4	1.7	1.9	**<0.001**
Milk and natural yogurt	28.0	53.8	1.2	2.1	0.8	1.7	1.6	2.4	**<0.001**
Coffee and tea	22.8	11.7	0.9	0.5	0.9	0.6	1.0	0.5	0.251
Fruit juice^c^	21.2	46.6	0.8	1.7	0.8	1.6	0.8	1.7	0.240
Fish	9.5	41.7	0.4	1.8	0.3	1.5	0.5	2.0	0.655
Cereal, seeds and nuts	3.4	29.5	0.1	0.9	0.1	1.2	0.1	0.6	0.370
**Processed culinary ingredients**	**303.4**	**127.3**	**12.4**	**4.6**	**12.2**	**4.4**	**12.6**	**4.9**	0.564
Sugars^d^	150.4	113.2	5.9	3.9	6.4	3.8	5.4	4.1	**<0.001**
Vegetable fat^e^	90.8	54.6	4.0	2.8	3.4	2.4	4.6	3.1	**<0.001**
Animal fat^f^	62.2	64.7	2.5	2.7	2.4	2.5	2.7	3.0	0.919
**Processed foods**	**133.8**	**174.6**	**5.2**	**6.2**	**5.5**	**6.5**	**4.8**	**5.9**	0.103
Pastries^g^	43.2	111.9	1.6	3.8	1.7	4.3	1.5	3.3	0.176
Processed breads, cookies and cakes^h^	41.1	78.8	1.7	3.1	1.7	3.0	1.6	3.2	0.287
Processed meats^i^	22.5	73.2	0.8	2.5	0.7	2.4	0.9	2.6	0.367
Fermented alcoholic beverages (beer and wine)	11.6	54.4	0.4	2.2	0.8	2.8	0.1	1.1	**<0.001**
Concentrated juice	7.7	24.4	0.3	1.0	0.3	1.1	0.3	0.9	0.876
Processed dairy products^j^	3.8	16.6	0.1	0.6	0.1	0.5	0.2	0.7	0.101
Canned food^k^	3.8	27.6	0.1	0.8	0.1	0.9	0.1	0.8	0.956
**Ultra-processed foods**	**452.8**	**326.3**	**17.7**	**10.8**	**17.0**	**10.4**	**18.4**	**11.1**	0.100
Industrialized breads, cookies and cereals^l^	110.9	164.8	4.4	6.3	3.3	4.9	5.6	7.4	**<0.001**
Industrialized pasta^m^	100.2	112.6	4.0	4.2	4.3	4.5	3.6	3.9	**0.031**
Candies^n^	62.3	114.5	2.4	4.4	1.6	3.3	3.2	5.2	**<0.001**
Sugary drinks (soda, juice and canned nectar and artificial soft drink)	52.6	76.0	1.9	2.6	2.4	3.0	1.5	2.2	**<0.001**
Industrialized sauces and condiment^o^ and margarine	46.7	49.7	1.8	1.8	1.8	1.8	1.9	1.9	0.708
Snacks and fried foods^p^	38.2	87.5	1.5	3.3	1.6	3.5	1.4	3.1	0.461
Sausages (ham, bacon and sausages)	33.6	76.0	1.3	2.7	1.5	2.9	1.0	2.4	**0.002**
Ultra-processed dairy products^q^	5.3	19.1	0.2	0.7	0.2	0.7	0.2	0.6	0.648
Distilled alcoholic drinks (*caipirinha*, brandy and spirit)	3.1	20.8	0.1	0.9	0.2	1.3	0.0	0.2	**<0.001**
**All foods**	**2561.0**	**883.1**	**100.0**	** -**	**100.0**	**-**	**100.0**	**-**	**-**

SD, Standard deviation. n, individuals number.

^a^ Like bananas, grape, guava, blackberry, watermelon, papaya, strawberry, apple, pear, pineapple, orange, tangerine, peach, persimmon, mango, lemon, avocado, lychee, plum, and fruit salad.

^b^ Like cucumber, tomato, lettuce, watercress, chard, peppers, radish, cabbage, gherkin, chayote, spinach, okra, zucchini, carrots, beets, green beans, taioba, broccoli, garlic, onions, chives, parsley, coriander, basil, and mint.

^c^ Natural or pulp juice, and coconut water.

^d^ White sugar, brown sugar, honey, and *rapadura*.

^e^ Sunflower oil, corn oil, soy oil, and olive oil.

^f^ Butter, cream, heavy cream, and lard.

^g^ Like jellies, *cocada*, *paçoca*, and condensed milk.

^h^ Like French bread, and French bread toast.

^i^ Like *dobradinha*, pork tail, dried meat, and crackling.

^j^ Minas cheese, rennet cheese and ricotta.

^k^ Canned sardines, green corn, palm hearts, and olives.

^l^ Like bread, sweet and salt cookies, wafer, stuffed cookie, cakes, breakfast cereal, pies and candies.

^m^ Like pizza, lasagna, sausage pie, noodles, and *escondidinho*.

^n^ Like chocolate, bonbon, whipped cream, bubble gum, lollipop, suspiro, *nhá benta*, caramels, *sorvete quente*, ice cream, popsicle, milkshake, and jelly.

^o^ Like mayonnaise, ketchup, condiments, industrialized tomato sauce, pepper sauce, *rosé* sauce and industrialized soups and broths.

^p^ Like pre-fried potatoes, breaded, microwave popcorn, chips, hamburgers and hot dogs.

^q^ Like creamy curd, parmesan cheese, *mozzarella* cheese, flavored yogurt.

* Mann-Whitney U test. n = 740.

The differences between the sexes were tested (Table 2), in order to verify if the individuals had differences in the *NOVA* group consumption that justified the stratification of the subsequent analyzes by sex. However, the caloric contribution according to the *NOVA* groups did not differ between sexes. About the food groups, we found that consumption tends to be higher in males, with the exception of some foods such as fruits, milk, vegetables and green condiment, vegetable fat, breads, cookies, cereals and candies, which was higher in females.

When assessing the adequacy of nutrient intake (Table 3), a high percentage of inadequate intake was identified for some dietary parameters such as calcium (100.0%), potassium (99.6%), fibers (98.2%), energy density (98.1%), vitamin B9 (91.8%), sodium (83.0%), vitamin C (74.2%), carbohydrate (67.6%), vitamin A (67.2%), protein (63.6%) and zinc (50.1%). However, a high percentage of consumption within the recommendation was identified for vitamin B1 (99.7%), cholesterol (99.6%), phosphorus (99.3%), iron (99.2%), copper (98.9%), vitamin B3 (98.0%), selenium (97.7%), vitamin E (97.0%), SFA (77.3%), vitamin B6 (59.6%), vitamin B2 (58.5%) and PUFA (55.4%).

**Table 3:** Proportion of individuals with adequate nutrient intake and the caloric contribution of minimally processed foods, processed culinary ingredients, processed foods and ultra-processed foods.

Proportion of individuals with adequate nutrient intake according to current recommendations		n	%	Minimally processed*		p-value†	Processed culinary ingredients*		p-value†	Processed foods*		p-value†	Ultra-processed foods*		p-value†
				50p	IQR		50p	IQR		50p	IQR		50p	IQR	
**Energy density** (kcal/g)^a^‡	Within the recommended	14	1.9	74.1	65.2–76.9	0.104	14.3	11.7–19.7	**0.031**	0.8	0.0–2.9	**0.047**	10.1	7.3–16.2	**0.041**
	Inadequate intake	726	98.1	66.2	56.7–74.7		11.9	8.9–15.2		3.2	0.0–7.5		15.6	9.8–24.3	
**Carbohydrate** (g)^b^	Within the recommended	240	32.4	67.1	56.5–75.2	0.268	12.7	10.2–16.3	**<0.001**	2.8	0.0–6.8	**0.019**	14.3	8.5–23.1	**0.044**
	Inadequate intake	500	67.6	65.7	57.0–74.3		11.6	8.5–14.4		3.5	0.0–8.3		15.7	10.1–24.7	
**Protein** (g)^b^	Within the recommended	269	36.4	64.8	54.2–72.6	**0.002**	12.2	8.8–14.9	0.742	3.3	0.0–7.2	0.909	17.6	11.4–27.9	**<0.001**
	Inadequate intake	471	63.6	66.9	58.4–75.7		11.8	9.0–15.4		3.1	0.0–7.4		14.6	8.9–22.6	
**Lipid** (g)^b^	Within the recommended	436	58.9	64.9	54.5–73.8	**<0.001**	11.7	8.6–14.5	**0.027**	3.7	0.1–8.4	**0.007**	17.0	10.6–26.4	**<0.001**
	Inadequate intake	304	41.1	68.3	59.5–75.9		12.2	9.6–16.2		2.5	0.0–6.9		14.1	8.5–20.5	
**SFA** (g)^c^	Within the recommended	572	77.3	65.4	55.6–74.2	**0.003**	11.9	8.9–15.0	0.073	3.5	0.0–7.9	**0.022**	16.4	10.2–25.3	**<0.001**
	Inadequate intake	168	22.7	68.6	59.5–76.5		12.2	9.8–16.6		2.4	0.0–6.7		13.7	7.4–19.3	
**PUFA** (g)^b^	Within the recommended	410	55.4	66.3	56.7–74.9	0.588	12.0	9.1–15.3	0.508	3.3	0.0–7.3	0.543	15.6	9.8–23.8	0.798
	Inadequate intake	330	44.6	66.3	57.0–74.7		11.9	8.9–15.1		3.0	0.0–8.3		15.4	9.5–24.5	
**Cholesterol** (mg)^b^	Within the recommended	737	99.6	66.3	57.0–74.8	0.676	12.0	9.0–15.2	0.188	3.1	0.0–7.4	0.149	15.5	9.7–24.2	0.866
	Inadequate intake	3	0.4	66.7	42.2–73.4		20.2	9.5–20.6		0.0	0.0–3.2		12.8	3.3–48.3	
**Fibers** (g)^b^	Within the recommended	13	1.8	73.8	68.5–76.7	**0.023**	14.0	12.0–18.0	0.089	0.0	0.0–3.9	0.053	10.1	5.5–16.6	**0.024**
	Inadequate intake	727	98.2	66.1	56.6–74.7		11.9	8.9–15.2		3.2	0.0–7.5		15.6	9.8–24.3	
**Vitamin A** (μg)^d^	Within the recommended	243	32.8	68.3	58.5–76.0	**0.010**	11.7	8.9–14.8	0.612	2.9	0.0–7.2	0.452	13.9	8.9–21.4	**0.005**
	Inadequate intake	497	67.2	65.3	55.8–73.9		12.1	9.0–15.3		3.3	0.0–7.5		16.6	10.1–25.2	
**Vitamin B1** (mg)^d^	Within the recommended	738	99.7	66.3	56.9–74.8	0.858	12.0	9.0–15.2	0.054	3.1	0.0–7.4	0.760	15.5	9.7–24.2	0.333
	Inadequate intake	2	0.3	67.3	59.9–74.8		18.7	17.4–19.9		3.6	0.0–7.1		10.4	5.3–15.6	
**Vitamin B2** (mg)^d^	Within the recommended	433	58.5	66.5	58.1–74.8	0.380	11.8	8.8–15.2	0.202	3.3	0.0–8.0	0.241	15.0	9.0–23.1	0.039
	Inadequate intake	307	41.5	65.9	55.4–74.8		12.3	9.4–15.2		2.9	0.0–6.9		16.9	10.2–25.9	
**Vitamin B3** (mg)^d^	Within the recommended	725	98.0	66.3	57.0–74.8	0.289	12.0	9.0–15.2	0.730	3.1	0.0–7.3	0.328	15.4	9.6–24.2	0.332
	Inadequate intake	15	2.0	62.6	53.4–70.2		12.9	7.6–14.4		5.8	0.2–9.8		18.7	12.5–28.3	
**Vitamin B6** (mg)^d^	Within the recommended	441	59.6	68.6	59.7–76.2	**<0.001**	11.6	8.8–14.7	**0.034**	2.8	0.0–7.2	0.290	13.8	8.2–21.3	**<0.001**
	Inadequate intake	299	40.4	63.0	52.8–71.2		12.4	9.3–15.9		3.6	0.0–8.3		19.1	11.9–28.1	
**Vitamin B9** (μg)^d^	Within the recommended	61	8.2	68.5	58.2–76.0	0.199	12.1	9.6–13.6	0.773	3.9	0.0–11.4	0.519	16.0	6.8–19.2	0.085
	Inadequate intake	679	91.8	66.1	56.5–74.6		11.9	8.9–15.3		3.1	0.0–7.3		15.4	9.8–24.5	
**Vitamin C** (mg)^d^	Within the recommended	191	25.8	67.4	58.3–75.2	0.127	12.3	9.7–15.8	**0.041**	2.0	0.0–6.9	**0.027**	14.7	9.1–21.7	0.105
	Inadequate intake	549	74.2	65.9	56.4–74.6		11.8	8.8–14.9		3.4	0.0–7.9		15.7	9.8–25.0	
**Vitamin E** (mg)^d^	Within the recommended	718	97.0	66.3	56.9–74.8	0.961	12.0	9.1–15.3	**0.003**	3.1	0.0–7.5	0.935	15.4	9.6–24.1	0.179
	Inadequate intake	22	3.0	64.9	58.6–75.6		8.4	5.9–12.4		3.4	0.3–6.4		18.3	13.0–27.7	
**Calcium** (mg)^d^	Inadequate intake	740	100.0	66.3	56.9–74.8	-	12.0	9.0–15.2	-	3.1	0.0–7.4	-	15.5	9.7–24.2	-
**Iron** (mg)^d^	Within the recommended	734	99.2	66.4	57.0–74.8	0.117	12.0	9.0–15.3	0.181	3.1	0.0–7.4	0.744	15.4	9.6–24.1	**0.014**
	Inadequate intake	6	0.8	56.6	48.5–65.3		9.8	6.2–13.2		1.4	1.1–13.5		29.1	18.9–39.5	
**Phosphorus** (mg)^d^	Within the recommended	735	99.3	66.4	57.1–74.8	**0.002**	12.0	9.0–15.2	0.424	3.1	0.0–7.4	**0.007**	15.4	9.6–24.1	**0.006**
	Inadequate intake	5	0.7	37.2	31.0–43.5		13.2	11.5–16.5		14.2	6.9–16.1		32.8	28.3–40.3	
**Potassium** (mg)^b^	Within the recommended	3	0.4	79.2	74.9–82.6	**0.032**	15.6	6.5–19.6	0.591	0.0	0.0–1.6	0.097	5.5	1.8–12.7	**0.047**
	Inadequate intake	737	99.6	66.2	56.9–74.7		12.0	9.0–15.2		3.2	0.0–7.4		15.5	9.8–24.2	
**Sodium** (mg)^b^	Within the recommended	126	17.0	63.5	54.5–72.5	**0.011**	12.5	9.4–14.6	0.625	3.3	0.0–7.2	0.516	19.3	12.4–27.5	**0.002**
	Inadequate intake	614	83.0	66.6	57.5–75.0		11.9	8.9–15.3		3.1	0.0–7.5		15.0	9.3–23.3	
**Selenium** (μg)^d^	Within the recommended	723	97.7	66.3	56.9–74.8	0.507	11.9	9.0–15.2	0.872	3.1	0.0–7.4	0.119	15.5	9.6–24.2	0.938
	Inadequate intake	17	2.3	64.3	59.2–71.9		13.4	7.5–16.5		6.2	2.0–13.3		14.6	10.6–22.3	
**Zinc** (mg)^d^	Within the recommended	369	49.9	66.3	57.2–74.9	0.281	11.6	8.7–15.0	0.071	3.2	0.0–7.3	0.648	15.4	9.5–23.6	0.555
	Inadequate intake	371	50.1	66.2	56.4–74.6		12.3	9.6–15.3		3.1	0.0–7.8		15.7	9.8–24.3	
**Copper** (μg)^d^	Within the recommended	732	98.9	66.2	56.8–74.8	0.454	11.9	9.0–15.2	0.750	3.1	0.0–7.4	0.512	15.4	9.7–24.2	0.846
	Inadequate intake	8	1.1	68.9	63.4–76.7		12.4	9.5–15.9		1.1	0.0–6.6		15.7	11.8–19.7	

n, individuals number. 50p, median. IQR, interquartile range (25^th^ percentile to 75^th^ percentile). SFA, saturated fatty acids. PUFA, polyunsaturated fatty acids.

^a^ WCRF & AICR (2007) [50]

^b^ WHO (2003) [51]

^c^ WHO (2018) [52]

^d^ FNB (1991–2011) [53]; Complete recommendations in S1 Table.

n = 740.

* Energy-adjusted and attenuated nutrients.

† Mann-Whitney U test.

‡ Calculated for the solid fraction of the diet, corresponding to the sum of calories from solid foods divided by the quantity in grams of these foods^a^.

When evaluating the proportion of individuals with adequate nutrient intake and the caloric contribution of each *NOVA* group, it can be observed that the farmers who have reached the recommendations of fibers, vitamins A and B6, phosphorus, and potassium were associated with higher energy consumption by minimally processed foods. In the group of processed culinary ingredients, higher consumption was associated with the proportion of individuals that presented adequacy of energy density, carbohydrates and vitamins C and E, and inadequacy of lipids and vitamin B6. Furthermore, the subjects who had inadequate consumption of energy density, carbohydrate, vitamin C, and phosphorus had a higher average caloric contribution from processed foods. Likewise, a higher contribution from ultra-processed foods was associated with inadequate consumption of energy density, carbohydrate, fiber, vitamins A and B6, iron, phosphorus, and potassium (Table 3).

The higher caloric contribution from minimally processed foods was associated with the lower caloric content of the diet (*P* = 0.003) and the greater macro and micronutrient content (protein, lipids, fiber, vitamins A and E, B vitamins, calcium, iron, phosphorus, potassium, sodium, selenium, zinc, and copper). After adjustments, farmers in the fourth consumption quartile of this food group showed lower energy consumption (β -0.16; *P*<0.001) and higher consumption of protein, lipid, SFA, cholesterol, and fiber. Likewise, the subjects with the highest consumption of minimally processed foods had the greater consumption of all 15 micronutrients analyzed (Table 4).

**Table 4:** Diet's nutritional profile according to the caloric contribution of the consumption of minimally processed foods by Brazilian farmers.

Nutrients*	1^st^ quartile		2^nd^ quartile		3^rd^ quartile		4^th^ quartile		p-value†	Crude‡		Ajusted‡	
	50p	IQR	50p	IQR	50p	IQR	50p	IQR		β	p-value	β	p-value
**Calorie** (kcal)	2137.9	1834.2–2502.0	2099.5	1795.6–2453.3	2017.1	1671.7–2380.8	1945.9	1706.3–2301.5	**0.003**	-0.19	**<0.001**	-0.16	**0.001**
**Kilojoules** (kJ)	8944.9	7674.2–10468.2	8784.2	7512.8–10264.7	8439.4	6994.4–9961.1	8141.7	7139.3–9629.4					
**Energy density** (kcal/g)§	1.9	1.7–2.1	1.9	1.7–2.1	1.9	1.6–2.2	1.9	1.7–2.2	0.997	0.00	0.988	-0.01	0.795
**Carbohydrate** (%)	49.9	42.1–59.9	49.7	41.1–59.6	52.7	41.5–62.1	52.8	44.7–60.9	0.177	0.08	0.115	0.06	0.286
**Protein** (%)	14.5	12.3–17.4	16.1	12.8–18.1	16.4	13.2–20.4	18.0	14.5–21.9	**<0.001**	0.31	**<0.001**	0.30	**<0.001**
**Lipid** (%)	29.6	25.8–36.6	32.0	27.1–38.2	32.7	27.6–39.7	33.1	28.8–39.8	**0.002**	0.18	**0.001**	0.17	**0.001**
**SFA** (%)	7.6	6.4–9.0	8.0	6.6–9.8	8.1	6.8–9.9	8.4	6.8–10.1	**0.034**	0.14	**0.006**	0.13	**0.013**
**PUFA** (%)	7.2	6.0–9.1	8.2	6.5–10.2	8.8	7.0–10.6	8.8	7.3–10.4	**<0.001**	0.25	**<0.001**	0.25	**<0.001**
**Cholesterol** (mg/1000 kcal)	108.7	88.0–134.0	117.0	94.2–147.3	120.1	101.2–151.1	130.3	106.7–163.2	**<0.001**	0.24	**<0.001**	0.22	**<0.001**
**Fibers** (g/1000 kcal)	9.7	7.6–12.7	11.0	8.6–13.9	11.1	8.9–14.6	12.6	10.3–16.3	**<0.001**	0.33	**<0.001**	0.32	**<0.001**
**Vitamin A** (μg/1000 kcal)	212.6	162.2–261.0	217.8	166.4–296.7	234.4	178.5–325.4	248.5	182.1–347.8	**<0.001**	0.18	**<0.001**	0.17	**0.001**
**Vitamin B1** (mg/1000 kcal)	0.8	0.7–1.0	0.9	0.7–1.1	0.9	0.8–1.2	1.1	0.8–1.3	**<0.001**	0.32	**<0.001**	0.31	**<0.001**
**Vitamin B2** (mg/1000 kcal)	0.5	0.4 - .6	0.5	0.4–0.6	0.5	0.4–0.7	0.5	0.4 - .7	**0.001**	0.17	**0.001**	0.14	**0.006**
**Vitamin B3** (mg/1000 kcal)	8.5	6.8–10.2	9.4	7.5–11.4	9.7	7.4–12.0	11.0	8.5–13.3	**<0.001**	0.33	**<0.001**	0.33	**<0.001**
**Vitamin B6** (mg/1000 kcal)	0.6	0.5–0.7	0.6	0.5–0.8	0.6	0.5–0.8	0.7	0.6 - .9	**<0.001**	0.33	**<0.001**	0.31	**<0.001**
**Vitamin B9** (μg/1000 kcal)	83.7	62.3–116.9	94.0	72.4–120.8	95.9	69.7–123.7	103.3	79.1–140.4	**<0.001**	0.21	**<0.001**	0.19	**<0.001**
**Vitamin C** (mg/1000 kcal)	42.8	28.6–63.1	43.3	31.0–64.1	47.8	32.7–68.5	48.6	31.0–71.5	0.209	0.13	**0.015**	0.15	**0.004**
**Vitamin E** (mg/1000 kcal)	10.4	8.4–13.7	11.6	8.9–15.4	12.6	9.5–15.9	12.9	10.5–15.8	**<0.001**	0.23	**<0.001**	0.22	**<0.001**
**Calcium** (mg/1000 kcal)	147.5	115.6–190.0	148.6	116.2–188.1	152.5	125.8–197.3	172.2	139.0–230.4	**<0.001**	0.19	**<0.001**	0.19	**<0.001**
**Iron** (mg/1000 kcal)	6.0	4.7–7.8	6.7	5.4–7.9	6.7	5.4–8.7	7.5	5.8–9.2	**<0.001**	0.16	**0.002**	0.14	**0.009**
**Phosphorus** (mg/1000 kcal)	418.3	338.0–524.7	458.5	364.7–533.9	473.4	379.2–583.8	525.1	430.3–640.9	**<0.001**	0.31	**<0.001**	0.30	**<0.001**
**Potassium** (mg/1000 kcal)	1091.2	883.9–1274.1	1151.3	962.5–1342.5	1195.6	995.9–1482.3	1294.4	1104.3–1590.9	**<0.001**	0.32	**<0.001**	0.29	**<0.001**
**Sodium** (mg/1000 kcal)	1118.0	940.0–1493.6	1272.7	968.1–1704.9	1305.4	986.7–1756.3	1394.4	1073.4–1847.7	**<0.001**	0.22	**<0.001**	0.22	**<0.001**
**Selenium** (μg/1000 kcal)	33.8	27.9–41.6	39.5	29.8–47.8	39.1	31.5–51.1	42.3	33.7–54.2	**<0.001**	0.29	**<0.001**	0.25	**<0.001**
**Zinc** (mg/1000 kcal)	3.6	2.9–4.6	3.9	3.2–4.8	4.1	3.1–5.0	4.1	3.4–5.2	**0.018**	0.16	**0.003**	0.17	**0.001**
**Copper** (μg/1000 kcal)	44.7	39.4–56.1	50.0	40.0–60.3	49.7	40.7–63.4	56.5	45.8–66.7	**<0.001**	0.29	**<0.001**	0.27	**<0.001**

n, individuals number. 50p, median. IQR, interquartile range (25^th^ percentile to 75^th^ percentile). β, regression coefficient. 95% IC, 95% confidence interval. SFA, saturated fatty acids. PUFA, polyunsaturated fatty acids.

n = 740.

* Energy-adjusted and attenuated nutrients.

† Kruskal-Wallis test.

‡ Linear regression. Adjusted Model by variables with p < 0.2 in the binary analyzes (according S2 Table).

§ Calculated for the solid fraction of the diet, corresponding to the sum of calories from solid foods divided by the quantity in grams of these foods [50].

When the caloric contribution from the processed culinary ingredients was greater, the caloric and energy density content of the diet was lower, and the contents of carbohydrate, lipid, PUFA, fibers, vitamins A, B1, B2, B3, B9, C and E, iron, potassium, sodium, selenium, and copper were higher. Likewise, after adjustments, individuals in the fourth quartile of processed culinary ingredients had lower caloric consumption (β -0.25; *P*<0.001) and energy density (β -0.17; *P* = 0.001). They also had a higher content of carbohydrate, lipid, SFA, PUFA, cholesterol, and fibers, as well as some micronutrients (vitamins A, B1, B2, B6, B9, C and E, potassium, sodium, selenium, zinc, and copper) compared to farmers in the first quartile (Table 5).

**Table 5:** Diet's nutritional profile according to the caloric contribution of the consumption of processed culinary ingredients by Brazilian farmers.

Nutrients*	1^st^ quartile		2^nd^ quartile		3^rd^ quartile		4^th^ quartile		p-value†	Crude‡		Ajusted‡	
	50p	IQR	50p	IQR	50p	IQR	50p	IQR		β	p-value	β	p-value
**Calorie** (kcal)	2221.3	1916.5–2623.1	2026.1	1697.2–2410.8	2032.1	1721.3–2453.3	1923.3	1665.3–2192.7	**<0.001**	-0.29	**<0.001**	-0.25	**<0.001**
**Kilojoules** (kJ)	9293.8	8018.6–10974.9	8477.2	7101.2–10086.8	8502.1	7202.1–10264.7	8047.1	6967.5–9174.3					
**Energy density** (kcal/g)§	2.0	1.8–2.2	1.9	1.7–2.1	1.9	1.7–2.2	1.8	1.6–2.1	**0.002**	-0.20	**<0.001**	-0.17	**0.001**
**Carbohydrate** (%)	45.9	37.7–54.8	51.8	42.1–60.1	52.2	43.4–61.1	55.8	48.5–66.1	**<0.001**	0.36	**<0.001**	0.32	**0.001**
**Protein** (%)	15.9	12.8–18.5	16.4	13.2–20.3	15.8	13.0–20.4	16.5	13.5–19.2	0.433	0.08	0.150	0.04	0.426
**Lipid** (%)	30.9	24.8–35.7	31.5	27.1–39.9	30.9	26.3–37.7	33.6	28.9–41.0	**<0.001**	0.22	**<0.001**	0.18	**<0.001**
**SFA** (%)	7.8	6.4–9.5	8.1	6.7–10.0	7.8	6.6–9.5	8.4	7.0–10.1	0.059	0.15	**0.004**	0.12	**0.016**
**PUFA** (%)	7.4	5.8–9.2	8.4	6.7–10.1	8.1	6.3–10.2	9.2	7.5–11.4	**<0.001**	0.34	**<0.001**	0.30	**<0.001**
**Cholesterol** (mg/1000 kcal)	119.3	90.7–141.1	120.4	98.9–149.7	114.7	94.6–146.0	120.5	99.2–155.2	0.312	0.14	**0.009**	0.11	**0.030**
**Fibers** (g/1000 kcal)	10.2	8.0–13.1	11.4	8.7–14.5	11.4	8.8–15.1	11.8	9.4–15.1	**0.002**	0.18	**0.001**	0.14	**0.006**
**Vitamin A** (μg/1000 kcal)	209.6	165.7–274.9	231.8	178.3–336.5	221.3	166.2–301.7	225.9	189.3–321.5	**0.048**	0.11	**0.030**	0.10	**0.049**
**Vitamin B1** (mg/1000 kcal)	0.8	0.7–1.1	0.9	0.7–1.2	0.9	0.7–1.2	1.0	0.8–1.2	**<0.001**	0.24	**<0.001**	0.21	**<0.001**
**Vitamin B2** (mg/1000 kcal)	0.5	0.4–0.6	0.5	0.4–0.7	0.5	0.4–0.6	0.6	0.5–0.7	**0.002**	0.18	**0.001**	0.15	**0.003**
**Vitamin B3** (mg/1000 kcal)	8.7	7.0–11.2	9.8	7.7–12.2	9.5	7.4–11.9	9.9	8.1–12.0	**0.019**	0.12	**0.016**	0.08	0.093
**Vitamin B6** (mg/1000 kcal)	0.6	0.5–0.8	0.7	0.5–0.8	0.6	0.5–0.8	0.7	0.5–0.8	0.064	0.13	**0.011**	0.10	**0.044**
**Vitamin B9** (μg/1000 kcal)	83.5	63.4–117.9	91.5	66.8–131.7	98.7	73.8–128.8	99.3	80.7–128.7	**0.002**	0.16	**0.003**	0.12	**0.016**
**Vitamin C** (mg/1000 kcal)	40.4	27.5–62.3	47.1	30.9–68.6	46.6	31.0–64.4	50.4	33.3–72.2	**0.009**	0.15	**0.004**	0.13	**0.009**
**Vitamin E** (mg/1000 kcal)	10.4	7.7–13.3	12.2	9.1–15.2	11.9	9.0–14.9	13.7	11.1–17.2	**<0.001**	0.38	**<0.001**	0.33	**<0.001**
**Calcium** (mg/1000 kcal)	155.2	121.3–209.1	154.2	128.3–204.3	151.5	125.4–194.5	154.0	124.1–195.2	0.935	-0.05	0.301	-0.09	0.081
**Iron** (mg/1000 kcal)	6.2	5.1–7.6	6.7	5.3–8.6	6.8	5.4–9.1	7.2	5.9–8.7	**0.001**	0.12	**0.018**	0.10	0.059
**Phosphorus** (mg/1000 kcal)	449.8	364.1–547.3	465.0	379.0–582.3	465.0	382.6–579.2	488.4	390.1–568.7	0.162	0.11	**0.043**	0.06	0.187
**Potassium** (mg/1000 kcal)	1104.3	931.5–1324.4	1196.3	970.0–1431.1	1143.4	964.2–1438.8	1277.3	1060.2–1504.1	**<0.001**	0.23	**<0.001**	0.19	**<0.001**
**Sodium** (mg/1000 kcal)	1135.8	885.4–1490.6	1305.4	986.7–1770.2	1248.8	959.5–1638.8	1402.4	1084.8–1836.6	**<0.001**	0.24	**<0.001**	0.22	**<0.001**
**Selenium** (μg/1000 kcal)	36.3	27.6–45.2	40.5	30.9–51.4	37.6	30.2–48.3	40.6	34.1–50.5	**<0.001**	0.22	**<0.001**	0.18	**<0.001**
**Zinc** (mg/1000 kcal)	3.8	3.0–4.9	4.1	3.3–5.0	3.8	3.1–4.9	4.2	3.2–5.0	0.120	0.11	**0.030**	0.10	**0.048**
**Copper** (μg/1000 kcal)	46.8	38.7–57.2	50.9	40.9–62.2	49.0	40.5–61.6	54.8	44.2–64.8	**<0.001**	0.22	**<0.001**	0.17	**0.001**

n, individuals number. 50p, median. IQR, interquartile range (25^th^ percentile to 75^th^ percentile). β, regression coefficient. 95% IC, 95% confidence interval. SFA, saturated fatty acids. PUFA, polyunsaturated fatty acids.

n = 740.

* Energy-adjusted and attenuated nutrients.

† Kruskal-Wallis test.

‡ Linear regression. Adjusted Model by variables with p < 0.2 in the binary analyzes (according S2 Table).

§ Calculated for the solid fraction of the diet, corresponding to the sum of calories from solid foods divided by the quantity in grams of these foods [50].

The higher caloric contribution from processed foods was associated with the greater caloric and density content of the diet and the lower content of the evaluated nutrients, except SFA, vitamin C, calcium and zinc, which did not show statistical differences. Still, after adjustments, individuals in the fourth quartile of processed foods had higher caloric intake (β 0.16, *P*<0.001) and energy density (β 0.13, *P* = 0.013), and lower consumption of 17 of the 23 macro and micronutrients evaluated (Table 6).

**Table 6:** Diet's nutritional profile according to the caloric contribution of the consumption of processed foods by Brazilian farmers.

Nutrients*	1^st^ quartile		2^nd^ quartile		3^rd^ quartile		4^th^ quartile		p-value†	Crude‡		Ajusted‡	
	50p	IQR	50p	IQR	50p	IQR	50p	IQR		β	p-value	β	p-value
**Calorie** (kcal)	1943.3	1674.0–2244.8	2078.4	1782.7–2448.4	2072.6	1812.1–2504.2	2127.7	1859.7–2510.4	**<0.001**	0.23	**<0.001**	0.16	**<0.001**
**Kilojoules** (kJ)	8130.7	7004.0–9392.2	8695.9	7458.6–10243.9	8671.6	7581.6–10477.7	8902.3	7781.0–10503.6					
**Energy density** (kcal/g)§	1.8	1.6–2.1	1.9	1.7–2.2	1.9	1.7–2.1	1.9	1.7–2.1	**0.008**	0.13	**0.012**	0.13	**0.013**
**Carbohydrate** (%)	54.9	47.4–62.2	50.4	41.5–58.9	49.7	40.5–59.6	48.5	39.7–59.3	**<0.001**	-0.20	**<0.001**	-0.14	**0.002**
**Protein** (%)	16.9	14.0–21.6	16.4	12.8–19.2	15.9	13.1–19.2	15.4	12.4–17.9	**0.003**	-0.17	**0.001**	-0.13	**0.008**
**Lipid** (%)	33.8	28.7–41.3	31.2	27.5–38.4	32.0	26.3–36.7	30.4	25.3–37.1	**0.001**	-0.18	**<0.001**	-0.12	**0.009**
**SFA** (%)	8.3	6.9–10.2	8.1	6.6–9.8	7.8	6.6–9.7	7.7	6.5–9.6	0.107	-0.12	**0.014**	-0.07	0.158
**PUFA** (%)	9.4	7.4–11.0	8.0	6.4–10.0	8.0	6.4–9.8	7.5	6.1–9.7	**<0.001**	-0.25	**<0.001**	-0.19	**<0.001**
**Cholesterol** (mg/1000 kcal)	123.7	101.7–158.7	117.8	100.2–144.6	117.0	93.9–149.9	117.5	89.8–137.8	**0.040**	-0.16	**0.001**	-0.12	**0.014**
**Fibers** (g/1000 kcal)	12.0	10.1–15.7	11.3	8.8–13.7	10.5	8.4–13.4	10.5	8.1–14.2	**<0.001**	-0.20	**<0.001**	-0.15	**0.003**
**Vitamin A** (μg/1000 kcal)	238.1	175.7–342.5	231.2	187.7–306.7	223.2	174.3–288.3	207.0	162.2–295.1	**0.022**	-0.14	**0.007**	-0.09	0.066
**Vitamin B1** (mg/1000 kcal)	1.1	0.8–1.3	0.9	0.7–1.1	0.8	0.7–1.2	0.8	0.7–1.1	**0.001**	-0.28	**<0.001**	-0.23	**<0.001**
**Vitamin B2** (mg/1000 kcal)	0.5	0.4–0.7	0.5	0.4–0.6	0.5	0.4–0.6	0.5	0.4–0.6	**0.007**	-0.14	**0.007**	-0.07	0.125
**Vitamin B3** (mg/1000 kcal)	10.3	8.3–13.0	9.8	7.4–11.9	9.1	7.1–11.1	8.7	7.0–11.1	**<0.001**	-0.22	**<0.001**	-0.18	**<0.001**
**Vitamin B6** (mg/1000 kcal)	0.7	0.5–0.9	0.6	0.5–0.8	0.6	0.5–0.7	0.6	0.5–0.8	**<0.001**	-0.21	**<0.001**	-0.16	**0.001**
**Vitamin B9** (μg/1000 kcal)	99.4	78.3–139.7	89.8	68.7–115.6	92.8	68.2–122.9	93.1	66.6–130.1	**0.03**	-0.09	0.081	-0.05	0.354
**Vitamin C** (mg/1000 kcal)	48.5	31.5–70.3	46.1	30.7–67.1	43.3	30.9–63.4	44.3	28.6–63.4	0.165	-0.12	**0.014**	-0.10	**0.041**
**Vitamin E** (mg/1000 kcal)	13.3	10.9–16.7	11.9	8.8–15.1	11.8	8.6–15.1	10.6	8.6–14.1	**<0.001**	-0.22	**<0.001**	-0.16	**0.001**
**Calcium** (mg/1000 kcal)	153.0	123.6–210.2	154.2	119.4–199.4	150.2	128.1–193.5	158.7	127.5–196.5	0.968	-0.03	0.591	0.02	0.686
**Iron** (mg/1000 kcal)	7.3	5.9–9.1	6.6	5.2–8.4	6.5	5.3–8.2	6.6	5.0–8.1	**<0.001**	-0.17	**0.001**	-0.11	**0.024**
**Phosphorus** (mg/1000 kcal)	492.2	403.5–618.8	466.4	366.6–562.1	458.5	379.0–557.9	449.0	356.8–537.3	**0.003**	-0.19	**<0.001**	-0.14	**0.004**
**Potassium** (mg/1000 kcal)	1243.4	1047.5–1569.6	1175.5	972.8–1398.1	1146.1	943.1–1384.3	1139.0	932.5–1345.6	**0.001**	-0.21	**<0.001**	-0.15	**0.002**
**Sodium** (mg/1000 kcal)	1445.0	1091.0–1877.8	1239.9	967.8–1582.4	1230.6	955.1–1585.1	1194.1	914.8–1597.6	**<0.001**	-0.19	**<0.001**	-0.15	**0.003**
**Selenium** (μg/1000 kcal)	43.9	33.7–54.0	39.0	31.4–47.5	36.0	28.5–46.7	36.6	28.6–45.8	**<0.001**	-0.23	**<0.001**	-0.17	**0.001**
**Zinc** (mg/1000 kcal)	4.1	3.2–5.3	3.8	3.0–4.9	3.9	3.1–4.7	3.9	3.1–4.9	0.141	-0.08	0.102	-0.04	0.362
**Copper** (μg/1000 kcal)	54.9	45.2–66.3	48.6	39.5–59.9	48.9	40.1–59.6	47.3	40.0–60.6	**<0.001**	-0.19	**<0.001**	-0.13	**0.008**

n, individuals number. 50p, median. IQR, interquartile range (25^th^ percentile to 75^th^ percentile). β, regression coefficient. SFA, saturated fatty acids. PUFA, polyunsaturated fatty acids.

n = 740.

* Energy-adjusted and attenuated nutrients.

† Kruskal-Wallis test.

‡ Linear regression. Adjusted Model by variables with p < 0.2 in the binary analyzes (according S2 Table).

§ Calculated for the solid fraction of the diet, corresponding to the sum of calories from solid foods divided by the quantity in grams of these foods [50].

In contrast to what was identified for the group of minimally processed foods, and similar to that found for processed foods, the higher caloric contribution from ultra-processed foods was associated with a greater caloric content of the diet (*P*<0.001) and lower content of all macro and micronutrients analyzed. This behavior was maintained after adjusted analyzes, in which the individuals in the highest consumption quartile of this food group had higher caloric consumption (β 0.17, *P*<0.001) and lower consumption of all 23 analyzed nutrients (Table 7).

**Table 7:** Diet's nutritional profile according to the caloric contribution of the consumption of ultra-processed foods by Brazilian farmers.

Nutrients*	1^st^ quartile		2^nd^ quartile		3^rd^ quartile		4^th^ quartile		p-value†	Crude‡		Ajusted‡	
	50p	IQR	50p	IQR	50p	IQR	50p	IQR		β	p-value	β	p-value
**Calorie** (kcal)	1923.7	1693.3–2279.5	2013.6	1644.9–2424.5	2078.2	1772.7–2447.9	2170.2	1908.0–2482.2	**<0.001**	0.21	**<0.001**	0.17	**<0.001**
**Kilojoules** (kJ)	8048.9	7084.7–9537.3	8424.9	6882.2–10143.9	8695.2	7417.0–10242.1	9080.0	7983.2–10385.5					
**Energy density** (kcal/g)§	1.9	1.6–2.2	1.9	1.6–2.2	1.9	1.6–2.1	1.9	1.8–2.1	0.290	0.08	0.137	0.08	0.154
**Carbohydrate** (%)	54.1	45.4–62.1	51.0	42.4–63.2	50.7	39.7–59.6	48.6	42.6–56.4	**0.007**	-0.15	**0.004**	-0.11	**0.038**
**Protein** (%)	16.9	13.8–21.6	16.8	13.8–20.7	16.1	13.2–19.6	14.4	12.4–17.1	**<0.001**	-0.29	**<0.001**	-0.26	**<0.001**
**Lipid** (%)	33.9	28.5–40.7	32.7	28.9–39.9	31.0	27.1–37.9	30.0	26.1–35.3	**<0.001**	-0.20	**<0.001**	-0.17	**0.001**
**SFA** (%)	8.5	6.8–10.4	8.4	7.1–9.8	7.8	6.4–9.8	7.7	6.5–8.9	**0.004**	-0.17	**0.001**	-0.14	**0.008**
**PUFA** (%)	9.0	7.4–11.0	9.0	7.2–10.5	8.0	6.6–9.9	7.1	5.9–9.1	**<0.001**	-0.32	**<0.001**	-0.31	**<0.001**
**Cholesterol** (mg/1000 kcal)	127.8	105.9–163.2	124.9	102.0–158.0	113.2	93.5–142.9	109.4	88.9–133.7	**<0.001**	-0.29	**<0.001**	-0.25	**<0.001**
**Fibers** (g/1000 kcal)	12.5	10.1–16.0	11.7	9.0–14.8	11.1	8.8–14.2	9.6	7.4–12.1	**<0.001**	-0.37	**<0.001**	-0.35	**<0.001**
**Vitamin A** (μg/1000 kcal)	255.6	187.8–344.7	233.6	177.7–327.9	210.9	166.7–271.6	211.9	158.3–257.5	**<0.001**	-0.21	**<0.001**	-0.16	**0.002**
**Vitamin B1** (mg/1000 kcal)	1.0	0.8–1.3	1.0	0.8–1.3	0.9	0.7–1.1	0.8	0.7–1.0	**<0.001**	-0.34	**<0.001**	-0.33	**<0.001**
**Vitamin B2** (mg/1000 kcal)	0.6	0.5–0.7	0.5	0.4–0.7	0.5	0.4–0.6	0.5	0.4–0.6	**<0.001**	-0.23	**<0.001**	-0.20	**<0.001**
**Vitamin B3** (mg/1000 kcal)	10.5	8.3–13.3	10.3	8.1–12.6	9.2	7.1–11.5	8.4	7.0–10.0	**<0.001**	-0.32	**<0.001**	-0.29	**<0.001**
**Vitamin B6** (mg/1000 kcal)	0.7	0.6–0.9	0.7	0.5–0.8	0.6	0.5–0.7	0.6	0.5–0.7	**<0.001**	-0.37	**<0.001**	-0.34	**<0.001**
**Vitamin B9** (μg/1000 kcal)	106.0	82.6–141.7	98.7	72.6–130.1	96.7	71.0–128.0	76.6	59.2–102.0	**<0.001**	-0.31	**<0.001**	-0.29	**<0.001**
**Vitamin C** (mg/1000 kcal)	49.1	31.0–67.2	47.4	32.7–69.7	47.3	29.9–69.0	39.8	28.6–56.5	**0.007**	-0.18	**0.001**	-0.19	**0.001**
**Vitamin E** (mg/1000 kcal)	13.3	10.8–16.7	12.6	10.1–15.9	11.5	8.6–14.9	10.1	8.1–13.7	**<0.001**	-0.32	**<0.001**	-0.32	**<0.001**
**Calcium** (mg/1000 kcal)	168.0	135.1–213.0	155.3	129.8–207.4	152.9	121.1–200.5	140.4	112.3–175.1	**<0.001**	-0.20	**<0.001**	-0.17	**0.001**
**Iron** (mg/1000 kcal)	7.6	6.1–9.2	6.7	5.4–8.9	6.7	5.3–8.2	5.7	4.7–7.3	**<0.001**	-0.20	**<0.001**	-0.16	**0.004**
**Phosphorus** (mg/1000 kcal)	506.0	418.0–632.5	488.9	392.2–602.5	463.5	382.6–557.9	411.5	335.2–502.8	**<0.001**	-0.34	**<0.001**	-0.30	**<0.001**
**Potassium** (mg/1000 kcal)	1321.4	1110.2–1605.0	1260.4	1022.4–1510.1	1146.1	943.7–1352.2	1057.6	870.0–1235.7	**<0.001**	-0.41	**<0.001**	-0.35	**<0.001**
**Sodium** (mg/1000 kcal)	1400.4	1073.4–1849.7	1378.8	1067.4–1791.9	1226.3	874.0–1551.2	1141.5	909.1–1492.0	**<0.001**	-0.22	**<0.001**	-0.21	**<0.001**
**Selenium** (μg/1000 kcal)	42.3	33.7–54.0	43.6	32.4–54.3	37.9	29.6–45.9	34.1	28.1–41.8	**<0.001**	-0.30	**<0.001**	-0.27	**<0.001**
**Zinc** (mg/1000 kcal)	4.2	3.4–4.9	4.2	3.2–5.2	3.9	3.1–5.1	3.6	2.9–4.4	**<0.001**	-0.20	**<0.001**	-0.17	**0.001**
**Copper** (μg/1000 kcal)	56.7	46.8–66.8	52.7	41.8–65.1	48.5	38.7–57.8	44.0	38.5–53.0	**<0.001**	-0.39	**<0.001**	-0.34	**<0.001**

n, individuals number. 50p, median. IQR, interquartile range (25^th^ percentile to 75^th^ percentile). β, regression coefficient. SFA, saturated fatty acids. PUFA, polyunsaturated fatty acids.

n = 740.

* Energy-adjusted and attenuated nutrients.

† Kruskal-Wallis test.

‡ Linear regression. Adjusted Model by variables with p < 0.2 in the binary analyzes (according S2 Table).

§ Calculated for the solid fraction of the diet, corresponding to the sum of calories from solid foods divided by the quantity in grams of these foods [50].

## Discussion

The major finding of this study was the clear identification of the differences between the nutritional profile of the food groups in this rural area, especially between the minimally processed, which had a higher nutritional content and less caloric intake, and the ultra-processed, which had a lower content of all macro and micronutrients analyzed as well as higher caloric content. These data demonstrate that the nutritional quality of food groups in this rural population is similar to that identified in other studies, in which ultra-processed foods have high energy density [16, 18–22, 25, 26, 29], micronutrient imbalance [15–17, 19, 20, 23–25, 28, 29], high content in sugar, saturated fat and trans fat and low in fiber [15–20, 22, 23, 25, 27–29].

In the Brazilian diet, ultra-processed foods showed 2.5 times more energy per gram, 2.0 times more free sugar, 1.5 times more fat in general and saturated fat and 8.0 times more trans fat, besides to having lower levels of fiber (3.0 times less), protein (2.0 times less) and potassium (2.5 times less) [23]. In addition, the increased participation of ultra-processed foods in the diet is inversely associated with the content of vitamins B3, B6, B12, D and E and copper, iron, phosphorus, magnesium, selenium and zinc [24]. The consumption of ultra-processed foods was also inversely associated with healthy pattern and directly associated with unhealthy pattern in the Brazilian diet [15].

When assessing the nutritional contents of the other food groups, we founded that nutritional quality due to the nutrient profile resulting from processed foods was similar to ultra-processed foods. The group of processed culinary ingredients had a higher content of carbohydrates and lipids and some nutrients, mainly due to the characteristics of the food items that make up this group, basically sources of fats and sugars. Processed culinary ingredients and minimally processed foods are directly related, since the are used in the kitchens to season and compose culinary preparations with them [48]. In this way, it is important to discuss that food processing, in itself, is not the problem, but rather the nature, extent and purpose of processing and the proportion of these food supplies in the human diet and their influence on world food patterns [49]. Food processing started as an adaptive procedure, developed in several ways to ensure edibility, palatability, microbiological safety of food and even increase the bioavailability of some nutrients [57, 58]. Diets restricted to unprocessed foods would be less diverse and even less safe, as foods become more available when processed by various harmless preservation methods [49]. Traditional cuisines established around the world are based on dishes and meals prepared with unprocessed and minimally processed foods, along with culinary ingredients and processed foods. It is concerned, then, with the large isocaloric exchange of the first three food groups for the ultra-processed group, and with the stages of ultra-processing of food, which leads to the addition of sugar, starch, sodium and hydrogenated and saturated fat [49, 57, 59].

This may also have reflected in the adequacy of nutrients to the fraction of the diet composed of each *NOVA* food group, which appeared to be better in the group of minimally processed foods and processed culinary ingredients. Furthermore, a low percentage of individuals was observed who met the current nutritional recommendations for many nutrients, such as calcium, potassium, sodium, zinc, vitamins A, B9 and C and fibers. This is in line with the data in the literature, in which there is a high prevalence of inadequacy in the intake of calcium, sodium, magnesium and vitamins A, C, D and E in Brazil, with this inadequacy being, in general, higher in the rural than in urban area [60].

It was also identified in this study that the caloric contribution of the diet from ultra-processed foods was slightly lower than the national average (17,7% *versus* 20.4%) [15]. This small difference is in accordance with the changes in the consumption profile in rural areas [33, 35–39], due to the low access to healthy food caused by its high cost [42], difficult access [41, 43], low education to provide healthy food choices [41], the understanding of foods produced only as merchandises [3], and the influences of rural labor structures [41].

In 2002–2003, the consumption of ultra-processed foods represented 20.0% of the caloric consumption of food in Brazil [8], increasing the share of ready-to-eat foods between 2002–2003 and 2008–2009 [14]. POF data 2008–2009 indicated that the average daily energy consumption of Brazilians was 1,866 kcal [15, 23], with 58.1% of unprocessed or minimally processed food, 10.9% of processed culinary ingredients, 10.6% of processed food and 20.4% of ultra-processed foods [15], a distribution similar to that found in the present study.

It is important to point out that the developed western countries present a greater caloric contribution coming from ultra-processed foods, as can be seen in the United States of America (60.0%) [11], United Kingdom (54.3%) [12] e Canada (45.0%) [13]. However, in developing countries this percentage is lower, as identified in Mexico (29.8%) [61], Chile (28.6%) [18] and here in Brazil (20.4%) [15].

A country that stands out for presenting a lower percentage of caloric contribution of this food group, even though it is a developed country, is France (35.9%) [17]. Such fact may be due to the presence of the traditional local food culture, which encourages home cooking, family meals and consumption of handmade products, proceeding as a protective factor for the high consumption of ultra-processed foods [17, 62].

A similar fact may occur in this study in rural areas, since many foods typical of Brazilian culture and local European colonization are maintained in this population [63]. This fact can be reinforced by the greater caloric contribution from minimally processed foods with processed culinary ingredients, producting especially culinary preparations such as breads, cakes, cookies, sweets and other homemade products. The rural area also shows higher adherence to traditional Brazilian food, with a higher consumption of beans and other legumes, rice, corn, cassava and yams [64], but also bread, leafy, milk, animal fat, margarine, sugar, cassava flour and coffee [65].

In addition, another fact that may contribute to the lower consumption of ultra-processed foods in this region is that the average price of *in natura* and minimally processed foods in Brazil (R$ 2.28/1000 kcal) is lower than the average price of ultra-processed foods (R$ 2.40/1000 kcal) [66]. However, a recent study identified that since the beginning of the 2000s the price of ultra-processed foods has undergone successive reductions in Brazil, becoming cheaper than processed foods, and it is predicted that unhealthy foods will become cheaper than healthy ones in 2026 [67]. As expected, the lowest caloric contribution of the farmers' diet provided by the group of processed food, as in Brazil (10.6%) [15]. Moreover, the consumption of minimally processed foods in this population was higher than the national one (64.7% *versus* 58.1%) [15] despite the low consumption of fruits and vegetables in rural regions [37, 38, 68].

When evaluating possible differences between the sexes, no relevant discrepancies were found in the caloric contribution of food groups and their food items. However, Bielemann et al. [25], in another Brazilian region, observed that the consumption of ultra-processed by women was slightly higher than in men. Nardocci et al. [13] found the opposite in the Canadian population, whose consumption of these foods was higher among young men.

Analizing the food groups, we found that consumption tends to be higher in males, with the exception of some foods such as fruits, milk, vegetables, bakery products and candies, which was higher in females. This is conforming to literature, since women adhere less to a traditional Brazilian food pattern [69], consuming these foods in their small meals, and eating more of fruits and vegetables in Brazil [70].

Regarding the possible limitations of the study, we can mention that the multiple R24h to collect food data may present bias in the interviewee's memory or difficulty in measuring the amount actually consumed, even with the help of photo albums of portions and utensils. Additionally, other micronutrients or phytochemicals can compose the variables that evaluate the nutritional profile of these food groups, however, only the nutrients presented in this study had their information available. Also, some nutrients such as sodium are not well estimated by food recall methods, and their interpretations are limited in this study. In the same way, the quantities of the processed culinary ingredients can be inaccurate, given the difficulty of accurately measuring the quantity of these items added to meals throughout the day. In addition, the micronutrient content in some typical foods in the region may have been underestimated, due to the lack of complete nutritional information on the recipes or the unavailability of all nutrients in the food composition table. Still, some culinary preparations that could not be disaggregated may have increased the percentage of caloric contribution from minimally processed foods.

Despite these possible biases, which are intrinsic to the methodology of analyzing food consumption in population studies, the evaluation of food consumption according to the degree and purpose of processing makes it possible to differentiate the quality of food, previously only grouped by its nutrient profile, in addition to assessing different characteristics intrinsic to eating behavior [8]. As well, to standardize their classification allows data comparison and monitoring of dietary changes over time [57, 71]. Besides, this method with multiple replications of R24h is a strong point of the study, since it allowed us to accurately evaluate the food items in a rural area.

Therefore, in this study it was identified that the group of foods with the greatest caloric contribution in the diet of these farmers was that of minimally processed foods, followed by ultra-processed foods, processed culinary ingredients, and processed foods. The caloric contribution from the consumption of ultra-processed foods is still slightly lower than the national average. However, measures aimed at delaying isocaloric exchanges between food groups must be carried out in order to maintain the local food culture, since ultra-processed foods showed worse nutritional levels. Furthermore, changes in eating habits in this rural region must be encouraged in order to increase the consumption of minimally processed foods, because it is precisely the group of foods that presented the best nutritional levels, especially of micronutrients in the diet.

## Supporting information

S1 Table Current recommendations of consumption of nutrients. SFA, saturated fatty acids. PUFA, polyunsaturated fatty acids. WCRF & AICR, World Cancer Research Fund & American Institute for Cancer Research. WHO, World Health Organization. FNB, Food and Nutrition Board. * Calculated for the solid fraction of the diet, corresponding to the sum of calories from solid foods divided by the quantity in grams of these foods [39].
(DOCX)

S2 Table Factors associated with food intake of nutrients for the selection of adjustment variables. *P*, p-value. n = 740. Mann-Whitney U test. * Kruskal-Wallis test. Values presented in median.
(DOCX)
