# Supplementary material for: Energy contribution of NOVA food groups and the nutritional profile of the Brazilian rural workers' diets
Source: PLoS One. 2020 Oct 28;15(10):e0240756. doi: 10.1371/journal.pone.0240756 (PMC7592810; doi:10.1371/journal.pone.0240756)
Supplement: S1 Table — SFA, saturated fatty acids. PUFA, polyunsaturated fatty acids. WCRF & AICR, World Cancer Research Fund & American Institute for Cancer Research. WHO, World Health Organization. FNB, Food and Nutrition Board. * Calculated for the solid fraction of the diet, corresponding to the sum of calories from solid foods divided by the quantity in grams of these foods [39]. (DOCX) [file pone.0240756.s001.docx]

**S1 Table. Current recommendations of consumption of nutrients**

| **Nutrient** | **Current recommendation** | **Reference** |
| --- | --- | --- |
| **Energy density*** | < 1.25 kcal/g | WCRF & AICR (2007) [50] |
| **Carbohydrate** | 55 to75% | WHO (2003) [51] |
| **Protein** | 10 to 15% | WHO (2003) [51] |
| **Lipid** | 20 to 35% | WHO (2003) [51] |
| **SFA** | < 10% | WHO (2018) [52] |
| **PUFA** | 6 to 10% | WHO (2003) [51] |
| **Cholesterol** | < 300 mg/day | WHO (2003) [51] |
| **Fibers** | > 25 g/day | WHO (2003) [51] |
| **Vitamin A** | Males: 625 to 3000 μg/day  Females: 500 to 3000 μg/day | FNB (1991-2011) [53] |
| **Vitamin B1** | Males: ≥ 1 mg/day  Females: ≥ 0.9 mg/day | FNB (1991-2011) [53] |
| **Vitamin B2** | Males: ≥ 1.1 mg/day  Females: ≥ 0.9 mg/day | FNB (1991-2011) [53] |
| **Vitamin B3** | Males: 12 to 35 mg/day  Females: 11 to 35 mg/day | FNB (1991-2011) [53] |
| **Vitamin B6** | Males: 1.1 to 100 mg/day  Females ≤ 50 years: 1.4 to 100 mg/day  Females > 50 years: 1.3 to 100 mg/day | FNB (1991-2011) [53] |
| **Vitamin B9** | 320 to 1000 μg/day | FNB (1991-2011) [53] |
| **Vitamin C** | Males: 75 to 2000 mg/day  Females: 60 to 2000 mg/day | FNB (1991-2011) [53] |
| **Vitamin E** | 12 to 1000 mg/day | FNB (1991-2011) [53] |
| **Calcium** | Males ≤ 50 years: 800 to 2500 mg/day  Males > 50 years: 800 to 2000 mg/day  Females ≤ 50 years: 800 to 2500 mg/day  Females > 50 years: 1000 to 2000 mg/day | FNB (1991-2011) [53] |
| **Iron** | Males: 6 to 45 mg/day  Females ≤ 50 years: 8.1 to 45 mg/day  Females > 50 years: 5 to 45 mg/day | FNB (1991-2011) [53] |
| **Phosphorus** | 580 to 4000 mg/day | FNB (1991-2011) [53] |
| **Potassium** | > 3500 mg/day | WHO (2018) [52] |
|  | < 2000 mg/day | WHO (2018) [52] |
| **Selenium** | 45 to 400 μg/day | FNB (1991-2011) [53] |
| **Zinc** | Males: 9.4 to 40 mg/day  Females: 6.8 to 40 mg/day | FNB (1991-2011) [53] |
| **Copper** | 700 to 10000 μg/day | FNB (1991-2011) [53] |

SFA, saturated fatty acids. PUFA, polyunsaturated fatty acids. WCRF & AICR, World Cancer Research Fund & American Institute for Cancer Research. WHO, World Health Organization. FNB, Food and Nutrition Board. * Calculated for the solid fraction of the diet, corresponding to the sum of calories from solid foods divided by the quantity in grams of these foods [50].
