# Supplementary material for: Energy contribution of NOVA food groups and the nutritional profile of the Brazilian rural workers' diets
Source: PLoS One. 2020 Oct 28;15(10):e0240756. doi: 10.1371/journal.pone.0240756 (PMC7592810; doi:10.1371/journal.pone.0240756)
Supplement: S2 Table — P, p-value. n = 740. Mann-Whitney U test. * Kruskal-Wallis test. Values presented in median. (DOCX) [file pone.0240756.s002.docx]

**S2 Table. Factors associated with food intake of nutrients for the selection of adjustment variables - *Part 1***

| **Variables** | **Calorie** | **Kilojoules** | **Energy density** (kcal/g) | **Carbohydrate** (%) | **Protein** (%) | **Lipid** (%) | **SFA** (%) | **PUFA** (%) | **Cholesterol** (mg/1000 kcal) | **Fibers** (g/1000 kcal) | **Vitamin A** (μg/1000 kcal) | **Vitamin B1** (mg/1000 kcal) | **Vitamin B2** (mg/1000 kcal) |
| --- | --- | --- | --- | --- | --- | --- | --- | --- | --- | --- | --- | --- | --- |
| **Sex** | ***P*<0.001** | ***P*<0.001** | *P=*0.092 | ***P*<0.001** | ***P*<0.001** | ***P*<0.001** | ***P*<0.001** | ***P*<0.001** | ***P*<0.001** | ***P*<0.001** | ***P*<0.001** | ***P*<0.001** | ***P*<0.001** |
| Male | 2276.4 | 9524.6 | 1.9 | 46.4 | 14.7 | 29.2 | 7.3 | 7.4 | 108.5 | 10.1 | 190.1 | 0.8 | 0.5 |
| Female | 1878.4 | 7859.3 | 1.9 | 56.1 | 17.2 | 35.7 | 8.8 | 9.3 | 130.9 | 12.5 | 260.6 | 1.0 | 0.6 |
| **Age group*** | ***P*<0.001** | ***P*<0.001** | ***P=*0.011** | ***P*<0.001** | ***P*<0.001** | ***P*<0.001** | ***P*<0.001** | ***P=*0.001** | ***P*<0.001** | ***P*<0.001** | ***P*<0.001** | ***P=*0.002** | ***P=*0.001** |
| Up to 29 years | 2192.7 | 9174.3 | 2.0 | 48.6 | 15.6 | 30.2 | 7.6 | 7.7 | 111.5 | 10.3 | 211.8 | 0.9 | 0.5 |
| 30 to 39 years | 2102.9 | 8798.7 | 1.9 | 49.8 | 14.8 | 31.2 | 7.7 | 8.2 | 114.8 | 10.7 | 216.0 | 0.9 | 0.5 |
| 40 to 49 years | 2006.9 | 8397.0 | 1.9 | 52.9 | 16.5 | 32.0 | 8.2 | 8.4 | 119.3 | 11.6 | 238.8 | 1.0 | 0.5 |
| 50 years or more | 1853.4 | 7754.6 | 1.8 | 56.1 | 19.1 | 35.8 | 9.2 | 9.1 | 140.6 | 12.1 | 247.8 | 1.0 | 0.6 |
| **Marital status*** | ***P=*0.001** | ***P=*0.001** | *P=*0.057 | ***P=*0.005** | ***P=*0.019** | ***P*<0.001** | ***P=*0.005** | ***P=*0.001** | ***P=*0.003** | ***P=*0.016** | *P=*0.091 | *P=*0.208 | *P=*0.393 |
| Single | 2278.8 | 9534.4 | 2.0 | 46.1 | 14.8 | 29.4 | 7.5 | 7.4 | 108.5 | 9.8 | 205.0 | 0.9 | 0.5 |
| Married/living with a partner | 2051.4 | 8582.9 | 1.9 | 51.2 | 16.1 | 31.9 | 8.0 | 8.2 | 119.4 | 11.3 | 225.7 | 0.9 | 0.5 |
| Divorced/separated/widowed | 1848.0 | 7731.9 | 1.9 | 56.0 | 17.1 | 36.5 | 9.2 | 9.4 | 132.8 | 13.1 | 251.6 | 1.0 | 0.5 |
| **Race/color** | *P=*0.445 | *P=*0.445 | ***P=*0.005** | *P=*0.256 | *P=*0.446 | *P=*0.456 | *P=*0.418 | *P=*0.436 | *P=*0.204 | *P=*0.917 | *P=*0.120 | *P=*0.580 | *P=*0.976 |
| White | 2039.4 | 8532.7 | 1.9 | 51.4 | 16.0 | 32.0 | 8.0 | 8.3 | 119.3 | 11.2 | 225.9 | 0.9 | 0.5 |
| Non-white | 2081.2 | 8707.9 | 1.8 | 50.6 | 17.2 | 31.3 | 7.8 | 7.9 | 116.5 | 10.9 | 216.4 | 0.9 | 0.5 |
| **Socioeconomic class*** | ***P=*0.001** | ***P=*0.001** | ***P=*0.034** | ***P=*0.001** | ***P=*0.007** | ***P=*0.009** | ***P=*0.144** | ***P=*0.001** | *P*=0.278 | ***P*<0.001** | ***P=*0.037** | ***P*<0.001** | ***P=*0.014** |
| A or B | 2223.8 | 9304.2 | 1.9 | 47.3 | 14.5 | 30.3 | 7.6 | 7.3 | 110.4 | 9.6 | 201.3 | 0.8 | 0.5 |
| C | 2082.0 | 8711.0 | 1.9 | 50.1 | 15.9 | 31.4 | 7.8 | 8.0 | 117.0 | 10.8 | 222.1 | 0.9 | 0.5 |
| D or E | 1992.2 | 8335.6 | 1.9 | 53.8 | 16.6 | 33.1 | 8.1 | 8.6 | 121.7 | 12.1 | 235.7 | 1.0 | 0.5 |
| **Schooling*** | ***P*<0.001** | ***P*<0.001** | *P=*0.081 | ***P*<0.001** | ***P*<0.001** | ***P*<0.001** | ***P=*0.001** | ***P*<0.001** | ***P*<0.001** | ***P*<0.001** | ***P=*0.005** | ***P*<0.001** | ***P*<0.001** |
| Less than 4 years | 1996.7 | 8354.1 | 1.9 | 52.9 | 16.6 | 33.0 | 8.2 | 8.6 | 123.7 | 11.8 | 231.8 | 1.0 | 0.5 |
| 4 to 8 years | 2191.1 | 9167.5 | 1.9 | 47.9 | 14.8 | 30.4 | 7.4 | 7.6 | 109.6 | 10.4 | 206.2 | 0.9 | 0.5 |
| More than 8 years | 2216.7 | 9274.6 | 2.0 | 47.6 | 14.8 | 29.7 | 7.7 | 7.1 | 110.9 | 10.2 | 225.7 | 0.8 | 0.5 |
| **Land bond** | ***P=*0.027** | ***P=*0.027** | ***P=*0.021** | *P=*0.067 | ***P=*0.013** | ***P=*0.036** | *P=*0.059 | *P=*0.114 | *P=*0.092 | ***P=*0.022** | *P=*0.247 | *P=*0.089 | *P=*0.349 |
| Owner | 2060.2 | 8619.8 | 1.9 | 51.1 | 15.8 | 31.5 | 7.8 | 8.1 | 116.9 | 11.0 | 221.5 | 0.9 | 0.5 |
| Non-owner | 2005.8 | 8392.3 | 1.8 | 52.2 | 16.8 | 32.7 | 8.3 | 8.6 | 122.4 | 12.4 | 233.3 | 0.9 | 0.5 |
| **Current type of production** | *P=*0.709 | *P=*0.709 | *P=*0.587 | *P=*0.432 | *P=*0.477 | *P=*0.703 | *P=*0.993 | *P=*0.265 | *P=*0.576 | *P=*0.593 | *P=*0.154 | *P=*0.891 | *P=*0.696 |
| Conventional | 2039.3 | 8532.4 | 1.9 | 51.1 | 16.0 | 32.2 | 8.0 | 8.3 | 118.2 | 11.3 | 222.1 | 0.9 | 0.5 |
| Non-conventional | 2073.3 | 8674.8 | 1.9 | 51.5 | 16.6 | 30.8 | 8.0 | 7.8 | 120.6 | 10.9 | 241.6 | 0.9 | 0.5 |
| **Type of worked crops*** | *P=*0.247 | *P=*0.247 | *P=*0.101 | *P=*0.057 | *P=*0.191 | *P=*0.574 | *P=*0.702 | ***P=*0.010** | *P=*0.568 | *P=*0.289 | *P=*0.645 | *P=*0.571 | *P=*0.970 |
| Temporary only | 2021.6 | 8458.2 | 1.9 | 52.2 | 15.6 | 32.7 | 7.9 | 8.5 | 119.3 | 11.4 | 221.0 | 0.9 | 0.5 |
| Permanent only | 2030.6 | 8496.0 | 1.9 | 54.2 | 17.2 | 31.3 | 8.1 | 8.0 | 125.3 | 11.4 | 234.4 | 0.9 | 0.5 |
| Temporary and permanent | 2078.0 | 8694.4 | 1.9 | 50.1 | 16.2 | 31.5 | 8.0 | 7.9 | 117.4 | 10.8 | 226.2 | 0.9 | 0.5 |
| **Workload (hours/week)** | ***P*<0.001** | ***P*<0.001** | ***P*<0.001** | ***P*<0.001** | ***P*<0.001** | ***P*<0.001** | ***P*<0.001** | ***P*<0.001** | ***P*<0.001** | ***P*<0.001** | ***P*<0.001** | ***P*<0.001** | ***P*<0.001** |
| Less than or equal to 40 hours | 1823.4 | 7629.2 | 1.8 | 56.4 | 17.2 | 37.0 | 8.5 | 9.3 | 126.4 | 12.6 | 233.6 | 1.0 | 0.6 |
| More than 40 hours | 2092.9 | 8756.6 | 1.9 | 49.9 | 15.7 | 31.3 | 7.8 | 8.0 | 115.8 | 10.8 | 220.5 | 0.9 | 0.5 |
| **Alcohol consumption** | ***P*<0.001** | ***P*<0.001** | *P=*0.997 | ***P*<0.001** | ***P=*0.001** | ***P*<0.001** | ***P=*0.010** | ***P*<0.001** | ***P=*0.005** | ***P=*0.004** | ***P*<0.001** | ***P=*0.018** | ***P=*0.001** |
| Non-drinking | 1964.8 | 8220.6 | 1.9 | 54.3 | 16.5 | 33.4 | 8.1 | 8.6 | 124.0 | 11.5 | 242.9 | 1.0 | 0.5 |
| Drinking | 2133.5 | 8926.4 | 1.9 | 47.9 | 15.7 | 30.8 | 7.8 | 7.8 | 114.4 | 10.8 | 203.4 | 0.9 | 0.5 |
| **Smoking** | ***P=***0.116 | *P=*0.116 | ***P=*0.088** | ***P=*0.008** | *P=*0.241 | *P=*0.223 | *P=*0.951 | ***P=*0.034** | *P=*0.839 | *P=*0.663 | ***P*<0.001** | *P=*0.363 | *P=*0.537 |
| Non-smoker | 2032.1 | 8502.1 | 1.9 | 52.0 | 16.0 | 32.2 | 8.0 | 8.3 | 118.5 | 11.2 | 229.4 | 0.9 | 0.5 |
| Current and past smoker | 2088.1 | 8736.4 | 1.8 | 47.3 | 16.5 | 31.1 | 8.1 | 7.7 | 120.5 | 11.3 | 194.7 | 0.9 | 0.5 |
| **Physical activity extra-field** | *P=*0.530 | *P=*0.530 | *P=*0.448 | *P=*0.596 | *P=*0.892 | *P=*0.189 | *P=*0.516 | *P=*0.099 | *P=*0.559 | *P=*0.712 | *P=*0.324 | *P=*0.598 | *P=*0.948 |
| No | 2030.6 | 8495.8 | 1.9 | 51.6 | 16.0 | 32.5 | 8.0 | 8.3 | 119.8 | 11.2 | 224.2 | 0.9 | 0.5 |
| Yes | 2083.1 | 8715.9 | 1.9 | 50.4 | 16.2 | 30.4 | 7.8 | 7.9 | 116.0 | 11.5 | 219.2 | 0.9 | 0.5 |
| **Screen time** | *P=*0.089 | *P=*0.089 | *P*=0.273 | *P=*0.064 | *P=*0.132 | *P=*0.267 | *P=*0.174 | *P=*0.586 | *P=*0.705 | *P=*0.070 | *P=*0.448 | ***P=*0.013** | *P=*0.147 |
| No sedentary leisure | 2020.9 | 8455.5 | 1.9 | 52.3 | 16.2 | 32.2 | 8.1 | 8.3 | 119.4 | 11.3 | 227.8 | 1.0 | 0.5 |
| With sedentary leisure | 2079.1 | 8699.0 | 1.9 | 50.3 | 15.9 | 31.5 | 7.9 | 8.2 | 117.5 | 10.9 | 221.3 | 0.9 | 0.5 |

*P,* p-value. n=740. Mann-Whitney U test. * Kruskal-Wallis test. Values presented in median.

**S2 Table. Factors associated with food intake of nutrients for the selection of adjustment variables - *Part 2***

| **Variables** | **Vitamin B3** (mg/1000 kcal) | **Vitamin B6** (mg/1000 kcal) | **Vitamin B9** (μg/1000 kcal) | **Vitamin C** (mg/1000 kcal) | **Vitamin E** (mg/1000 kcal) | **Calcium** (mg/1000 kcal) | **Iron** (mg/1000 kcal) | **Phosphorus** (mg/1000 kcal) | **Potassium** (mg/1000 kcal) | **Sodium** (mg/1000 kcal) | **Selenium** (μg/1000 kcal) | **Zinc** (mg/1000 kcal) | **Copper** (μg/1000 kcal) |
| --- | --- | --- | --- | --- | --- | --- | --- | --- | --- | --- | --- | --- | --- |
| **Sex** | ***P<*0.001** | ***P<*0.001** | ***P<*0.001** | ***P<*0.001** | ***P<*0.001** | ***P<*0.001** | ***P<*0.001** | ***P<*0.001** | ***P<*0.001** | ***P<*0.001** | ***P<*0.001** | ***P<*0.001** | ***P<*0.001** |
| Male | 8.7 | 0.6 | 84.3 | 39.8 | 10.7 | 137.9 | 6.2 | 422.2 | 1091.9 | 1175.5 | 36.4 | 3.6 | 45.8 |
| Feminine | 10.2 | 0.7 | 106.0 | 54.5 | 13.3 | 176.4 | 7.5 | 516.9 | 1265.3 | 1414.1 | 41.9 | 4.3 | 55.4 |
| **Age group*** | ***P<*0.001** | ***P<*0.001** | ***P<*0.001** | ***P=*0.040** | ***P=*0.012** | ***P<*0.001** | ***P<*0.001** | ***P<*0.001** | ***P<*0.001** | ***P=*0.004** | ***P=*0.004** | ***P<*0.001** | ***P<*0.001** |
| Up to 29 years | 9.1 | 0.6 | 88.6 | 42.5 | 11.4 | 144.4 | 6.4 | 447.0 | 1091.2 | 1247.3 | 37.7 | 3.9 | 47.1 |
| 30 to 39 years | 9.2 | 0.6 | 90.1 | 45.9 | 11.7 | 150.0 | 6.4 | 442.9 | 1176.1 | 1193.9 | 37.3 | 3.7 | 48.9 |
| 40 to 49 years | 9.7 | 0.7 | 105.8 | 48.5 | 12.2 | 159.0 | 7.2 | 479.0 | 1208.9 | 1251.4 | 38.6 | 4.0 | 50.6 |
| 50 years or more | 10.8 | 0.7 | 102.0 | 48.0 | 13.0 | 179.2 | 7.6 | 547.0 | 1336.7 | 1417.0 | 42.2 | 4.7 | 56.4 |
| **Marital status*** | *P=*0.367 | *P=*0.058 | *P=*0.247 | *P=*0.087 | ***P=*0.005** | ***P=*0.013** | ***P=*0.029** | ***P=*0.028** | ***P=*0.013** | ***P=*0.031** | *P=*0.280 | *P=*0.099 | *P=*0.067 |
| Single | 9.0 | 0.6 | 84.5 | 37.9 | 10.8 | 135.7 | 5.8 | 419.1 | 1112.1 | 1107.6 | 37.5 | 3.8 | 45.0 |
| Married/living with a partner | 9.4 | 0.6 | 95.4 | 46.8 | 11.8 | 155.7 | 6.7 | 467.1 | 1195.3 | 1272.6 | 38.7 | 3.9 | 50.1 |
| Divorced/separated/widowed | 10.2 | 0.7 | 93.7 | 48.8 | 13.7 | 156.6 | 7.7 | 490.4 | 1237.0 | 1404.2 | 41.6 | 4.4 | 53.4 |
| **Race/color** | *P=*0.712 | *P=*0.757 | *P=*0.622 | *P=*0.660 | *P=*0.838 | *P=*0.795 | *P=*0.329 | *P=*0.553 | *P=*0.243 | *P=*0.655 | *P=*0.450 | *P=*0.493 | *P=*0.294 |
| White | 9.5 | 0.6 | 93.6 | 46.1 | 11.8 | 153.3 | 6.7 | 465.0 | 1186.4 | 1261.1 | 38.6 | 3.9 | 50.0 |
| Non-white | 9.8 | 0.7 | 100.5 | 45.2 | 12.2 | 156.1 | 7.0 | 491.5 | 1178.2 | 1247.0 | 40.5 | 4.1 | 48.7 |
| **Socioeconomic class*** | ***P=*0.001** | ***P=*0.023** | ***P=*0.002** | *P=*0.422 | ***P=*0.046** | ***P=*0.030** | ***P<*0.001** | ***P<*0.001** | ***P<*0.001** | ***P=*0.182** | ***P=*0.001** | *P=*0.233 | ***P<*0.001** |
| A or B | 8.9 | 0.6 | 92.0 | 40.1 | 10.9 | 144.6 | 6.0 | 407.2 | 1020.0 | 1344.7 | 34.9 | 3.8 | 43.4 |
| C | 9.1 | 0.6 | 90.1 | 47.9 | 11.8 | 152.4 | 6.5 | 457.1 | 1172.7 | 1238.2 | 37.9 | 3.9 | 48.2 |
| D or E | 10.0 | 0.7 | 102.4 | 45.0 | 12.4 | 159.3 | 7.1 | 487.8 | 1232.3 | 1320.1 | 40.5 | 4.1 | 53.9 |
| **Schooling*** | ***P<*0.001** | ***P<*0.001** | ***P<*0.001** | ***P=*0.022** | ***P<*0.001** | ***P=*0.016** | ***P<*0.001** | ***P<*0.001** | ***P<*0.001** | ***P=*0.009** | ***P<*0.001** | ***P<*0.001** | ***P<*0.001** |
| Less than 4 years | 9.9 | 0.7 | 98.4 | 47.8 | 12.5 | 157.0 | 7.1 | 488.1 | 1243.4 | 1314.1 | 40.6 | 4.1 | 52.4 |
| 4 to 8 years | 8.8 | 0.6 | 92.4 | 40.5 | 10.9 | 148.6 | 6.2 | 444.0 | 1107.2 | 1192.3 | 37.7 | 3.7 | 44.6 |
| More than 8 years | 7.9 | 0.6 | 84.2 | 47.7 | 10.5 | 152.2 | 6.2 | 424.5 | 1090.9 | 1197.2 | 33.6 | 3.9 | 46.2 |
| **Land bond** | *P=*0.081 | *P=*0.307 | *P=*0.101 | *P=*0.555 | *P=*0.457 | ***P=*0.035** | *P=*0.153 | ***P=*0.008** | *P=*0.087 | *P=*0.650 | *P=*0.292 | ***P=*0.045** | *P=*0.055 |
| Owner | 9.4 | 0.6 | 93.0 | 45.8 | 11.8 | 152.0 | 6.7 | 458.5 | 1175.0 | 1266.8 | 38.4 | 3.9 | 49.2 |
| Non-owner | 9.9 | 0.7 | 100.0 | 46.5 | 12.3 | 160.1 | 7.0 | 489.1 | 1231.5 | 1250.9 | 39.3 | 4.1 | 53.0 |
| **Current type of production** | *P=*0.892 | *P=*0.155 | *P=*0.624 | *P=*0.606 | *P=*0.952 | *P=*0.736 | *P=*0.608 | *P=*0.851 | *P=*0.730 | ***P<*0.001** | *P=*0.947 | *P=*0.783 | *P=*0.742 |
| Conventional | 9.4 | 0.6 | 94.8 | 46.5 | 11.8 | 153.1 | 6.7 | 464.4 | 1189.5 | 1247.3 | 38.7 | 3.9 | 49.9 |
| Non-conventional | 9.9 | 0.7 | 94.8 | 42.0 | 12.4 | 155.8 | 7.4 | 478.4 | 1160.6 | 1599.3 | 38.8 | 4.1 | 49.8 |
| **Type of worked crops*** | *P=*0.677 | *P=*0.378 | *P=*0.416 | *P=*0.171 | *P=*0.071 | ***P=*0.049** | *P=*0.748 | *P=*0.336 | *P=*0.521 | *P=*0.762 | *P=*0.863 | ***P=*0.047** | *P=*0.172 |
| Temporary only | 9.8 | 0.6 | 96.3 | 46.3 | 12.2 | 149.1 | 6.7 | 462.3 | 1177.7 | 1254.9 | 38.4 | 3.8 | 50.7 |
| Permanent only | 9.8 | 0.7 | 106.7 | 52.8 | 11.5 | 163.1 | 7.2 | 516.9 | 1234.9 | 1233.8 | 38.4 | 4.5 | 53.5 |
| Temporary and permanent | 9.3 | 0.7 | 92.7 | 44.3 | 11.7 | 158.7 | 6.7 | 463.0 | 1182.2 | 1282.8 | 38.8 | 4.0 | 48.9 |
| **Workload (hours/week)** | ***P<*0.001** | ***P<*0.001** | ***P=*0.003** | ***P=*0.008** | ***P<*0.001** | ***P<*0.001** | ***P<*0.001** | ***P<*0.001** | ***P<*0.001** | ***P=*0.033** | ***P=*0.001** | ***P<*0.001** | ***P<*0.001** |
| Less than or equal to 40 hours | 10.3 | 0.7 | 106.9 | 49.9 | 13.3 | 170.8 | 7.7 | 516.2 | 1287.0 | 1386.5 | 42.4 | 4.4 | 56.6 |
| More than 40 hours | 9.2 | 0.6 | 92.4 | 44.5 | 11.6 | 150.0 | 6.6 | 457.7 | 1162.6 | 1247.3 | 38.3 | 3.8 | 48.7 |
| **Alcohol consumption** | ***P=*0.005** | *P=*0.094 | *P=*0.079 | ***P=*0.003** | ***P<*0.001** | ***P<*0.001** | ***P=*0.004** | ***P=*0.003** | ***P<*0.001** | ***P=*0.028** | ***P=*0.021** | ***P=*0.008** | ***P<*0.001** |
| Non-drinking | 9.9 | 0.7 | 96.7 | 48.7 | 12.4 | 160.7 | 7.0 | 483.8 | 1208.4 | 1309.5 | 40.1 | 4.1 | 52.4 |
| Drinking | 9.0 | 0.6 | 92.1 | 42.3 | 11.3 | 147.2 | 6.4 | 452.3 | 1151.3 | 1226.3 | 37.7 | 3.8 | 47.1 |
| **Smoking** | *P=*0.692 | *P=*0.441 | *P=*0.678 | ***P<*0.001** | *P=*0.078 | *P=*0.518 | *P=*0.883 | *P=*0.190 | *P=*0.752 | *P=*0.574 | *P=*0.999 | *P=*0.148 | *P=*0.948 |
| Non-smoker | 9.5 | 0.6 | 94.1 | 47.4 | 12.0 | 154.0 | 6.7 | 462.6 | 1182.5 | 1272.7 | 38.8 | 3.9 | 49.6 |
| Current and past smoker | 9.5 | 0.6 | 96.7 | 37.7 | 11.3 | 152.9 | 6.8 | 478.8 | 1218.8 | 1247.1 | 38.3 | 4.3 | 50.9 |
| **Physical activity extra-field** | *P=*0.154 | *P=*0.639 | *P=*0.820 | *P=*0.823 | *P=*0.209 | *P=*0.459 | *P=*0.910 | *P=*0.854 | *P=*0.868 | *P=*0.451 | *P=*0.511 | *P=*0.675 | *P=*0.546 |
| No | 9.4 | 0.6 | 93.6 | 45.4 | 11.9 | 154.3 | 6.7 | 464.9 | 1185.4 | 1278.0 | 38.9 | 3.9 | 50.4 |
| Yes | 10.0 | 0.7 | 96.6 | 47.6 | 11.6 | 149.7 | 6.8 | 477.1 | 1185.1 | 1215.9 | 37.1 | 4.0 | 47.8 |
| **Screen time** | *P=*0.244 | *P=*0.240 | *P=*0.307 | *P=*0.478 | *P=*0.801 | *P=*0.056 | ***P=*0.007** | *P=*0.081 | *P=*0.120 | ***P=*0.007** | *P=*0.127 | *P=*0.987 | *P=*0.460 |
| No sedentary leisure | 9.6 | 0.7 | 95.5 | 47.1 | 11.9 | 159.7 | 7.0 | 477.1 | 1202.0 | 1318.4 | 39.3 | 3.9 | 50.1 |
| With sedentary leisure | 9.3 | 0.6 | 93.8 | 45.5 | 11.8 | 149.9 | 6.5 | 452.6 | 1166.0 | 1226.3 | 38.0 | 4.0 | 49.4 |

*P,* p-value. n=740. Mann-Whitney U test. * Kruskal-Wallis test. Values presented in median.
